# Accessing human selenoproteins through chemical protein synthesis†
†Electronic supplementary information (ESI) available. See DOI: 10.1039/c6sc04123j



**DOI:** 10.1039/c6sc04123j

**Published:** 2016-11-01

**Authors:** L. Dery, P. Sai Reddy, S. Dery, R. Mousa, O. Ktorza, A. Talhami, N. Metanis

**Affiliations:** a Institute of Chemistry , The Hebrew University of Jerusalem , Edmond J. Safra, Givat Ram , Jerusalem 91904 , Israel . Email: Metanis@mail.huji.ac.il

## Abstract

The human body contains 25 selenoproteins, which contain in their sequence the twenty-first encoded amino acid, selenocysteine. About a dozen of these proteins remain functionally uncharacterized or poorly studied. Challenges in accessing these selenoproteins using traditional recombinant expressions have prevented biological characterization thus far. Chemical protein synthesis has the potential to overcome these hurdles. Here we report the first total chemical syntheses of two human selenoproteins, selenoprotein M (SELM) and selenoprotein W (SELW). The synthesis of the more challenging protein SELM was enabled using recent advances in the field of selenocysteine chemistry. This approach allows the preparation of selenoproteins in milligram quantities and in homogenous form, which should open new horizons for future studies to pursue a fuller biological understanding of their role in health and disease.

## Abstract 2

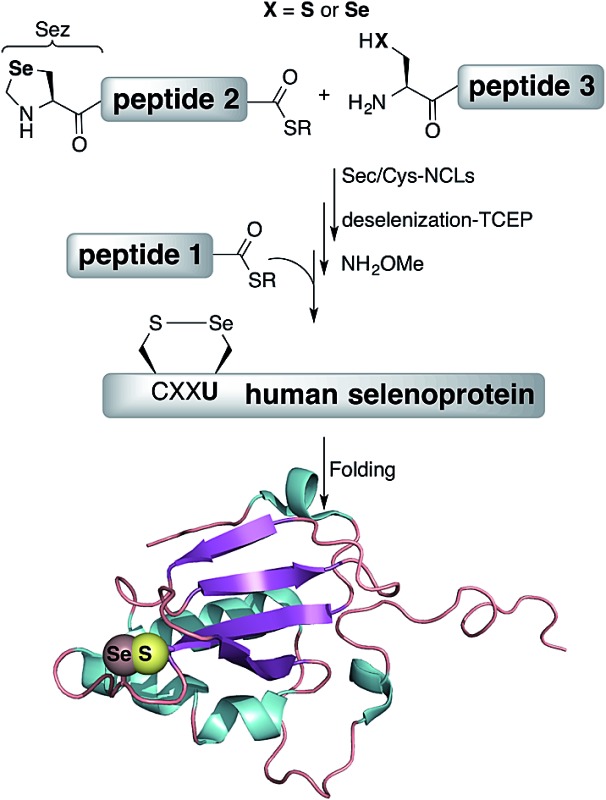
The human body contains 25 selenoproteins, but challenges in their preparations have prevented biological characterizations thus far. Here we report the first total chemical syntheses of two human selenoproteins, selenoprotein M (SELM) and selenoprotein W (SELW).

## Introduction

Selenium is an essential trace nutrient for human health,^1^ which is present mainly in the form of selenocysteine (Sec, U). Sec is the twenty-first proteinogenic amino acid, and is commonly found in the active site of selenoproteins.^2–4^ The human body contains 25 selenoproteins,^5^ yet the biological function of many of these proteins remains unclear or poorly studied.

Sec's codon, UGA, is normally a “stop codon”, which signals for truncation.^1–4,6^ Therefore, Sec is incorporated co-translationally into selenoproteins by the suppression of UGA. This highly regulated process requires multiple components, including a characteristic mRNA stem-loop structure called selenocysteine insertion sequence (SECIS) element, a dedicated Sec-specific elongation factor (EFSec), a unique tRNA (tRNA^Sec^), SECIS-binding protein 2 (SBP2) and other factors (*e.g.* in eukaryotes) to guarantee translation fidelity.^7–9^ For these reasons, it is challenging to prepare sufficient amounts of selenoproteins in homogenous forms using traditional recombinant expression systems, despite recent developments in the field.^10–13^

Because the recombinant expression of wild type selenoproteins is inefficient,^14^ many research groups study Sec-to-Cys mutants instead. Although these studies can provide valuable insights into selenoproteins' structures and functions, they are executed on mutant proteins and not the natural forms. Moreover, many of the Sec-to-Cys mutants exhibited decreased catalytic activity by up to three orders of magnitude.^15–17^ To this end, in order to understand the function of natural selenoproteins such as human selenoprotein M (SELM) and selenoprotein W (SELW), studies on their Sec-containing forms are essential.^18^

Chemical protein synthesis or semi-synthesis (CPS) is a powerful approach allowing the preparation of proteins with sequences beyond the 20 canonical amino acids, including selenoproteins.^19–26^ CPS is based mainly on solid-phase peptide synthesis (SPPS)^27^ and chemoselective ligation reactions,^28–32^ for example native chemical ligation (NCL).^28^ Using this technology, it is possible to prepare (seleno)proteins of up to ∼200 amino acids.^29,31,33–40^ In principle, even larger (seleno)proteins can be prepared using expressed protein ligation (EPL).^19,22–25,41,42^ CPS is particularly relevant for human selenoproteins, many of which are fewer than 200 amino acids in length (Table S1†).^5^

**Scheme 1:**
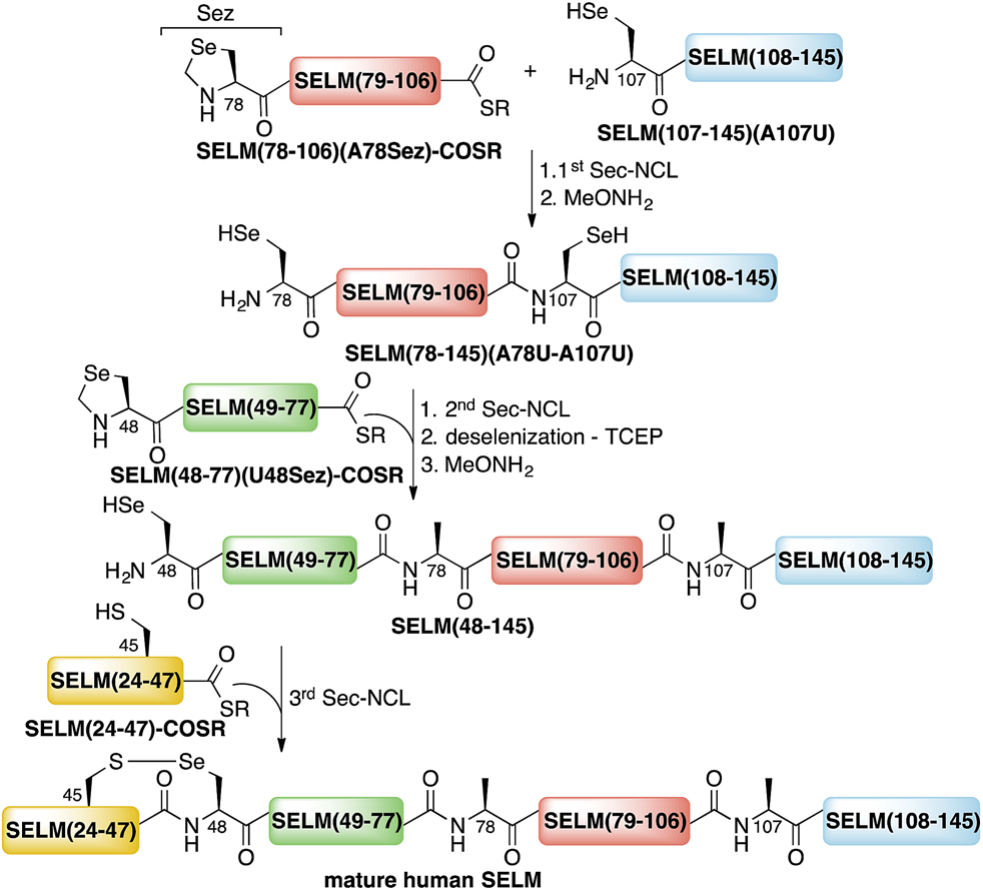
Total chemical synthesis approach for mature human SELM based on four segments with three sequential Sec-NCL reactions, utilizing Sez and a deselenization reaction.

**Scheme 2:**
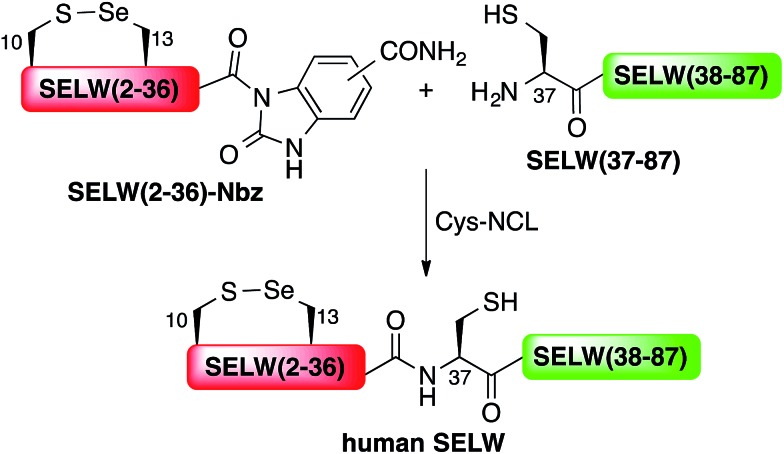
Total chemical synthesis of wildtype human SELW.

Here, we present the first total chemical syntheses of two human selenoproteins. Our SELM synthesis is based on four segments with three sequential Sec-NCL reactions, and utilizes a protected form of Sec, selenazolidine (Sez),^43^ as well as a deselenization reaction (Scheme 1) that we and others have recently developed.^34,44,45^ The shortest member of the selenoprotein family, SELW, was prepared from two peptide segments with a single Cys-NCL reaction (Scheme 2).

Human SELM, an endoplasmic reticulum (ER) selenoprotein, is expressed in many tissues in the body, but is most abundant in the brain,^46^ suggesting an important role in the nervous system. The 145 amino acid protein can be viewed in three parts (Fig. S1†). The first segment, an N-terminal ER signal peptide (1–23), is cleaved upon translocation,^46^ making the mature SELM 122 amino acids in length. The second segment, a Trx-fold adjacent to the signal peptide, contains the active site redox motif ^45^CXX^48^U, which is similar to CXXC in the thioredoxin (Trx) superfamily^47,48^ and suggests a role as a thiol-disulfide-like oxidoreductase. This motif has also been identified in other human selenoproteins, including SELW, SELH, SELT, SELV and SEP15 (CXU).^5,49,50^ Indeed, NMR structural analyses of mutant mouse SelM(U48C) and wildtype fruit-fly Sep15 (which is not a selenoprotein, CXC motif) suggested that they are homologues of one another, and form a distinct selenoprotein family within the Trx superfamily.^51,52^ The third segment in SELM is the C-terminal ER retention sequence (HADL) that ensures the protein remains in the ER.^53^

The other selenoprotein investigated in this study, SELW, is a small cytosolic protein that was found to be absent from muscles in lambs and calves suffering from white muscle disease (WMD).^54–58^ It is highly conserved in mammals, and is one of the most highly expressed selenoproteins.^59,60^ The ^10^CXX^13^U motif in the N-terminus of SELW is similar to that of SELM and other members of the Trx superfamily (*vide supra*). SELW was also suggested to have a fundamental role in the cell cycle,^61–64^ and the interaction between SELW and 14-3-3 proteins was confirmed by high-resolution NMR studies using a double mutant mouse SelW(C10S–U13C).^60^ Despite all the accumulated data, no definitive biological function has been assigned to SELW due to the challenges in accessing selenoproteins.

Here we show for the first time the total chemical syntheses of the two human selenoproteins, SELM and SELW. The synthesis of the more challenging protein SELM was enabled using recent advances in the field of selenocysteine chemistry and represents the first Sec-driven multistep ligations to create a protein. This approach allow the preparation of human and other natural (or unnatural) selenoproteins in milligram quantities and in homogenous form, which should allow future studies to pursue a fuller biological understanding of their role in health and disease.

## Results and discussion

The synthesis of mature human SELM is challenging, as it is 122 amino acids long and contains both Cys and Sec residues in the ^45^CXX^48^U motif. Because these residues are located non-strategically into the protein sequence, only one of them can be useful for CPS using NCL reaction. Therefore, we decided to prepare SELM from four segments with three sequential NCL reactions^65^ at the following ligation sites: Asn106–Ala107, Gly77–Ala78, and Gly47–Sec48 (Scheme 1). To allow for sequential ligations, we temporary substituted Ala107 with Sec, and both Ala78 and Sec48 with Sez, which we recently developed as a useful tool for the chemical synthesis of proteins with non-strategically positioned Cys (or Sec) residues.^43^ Additionally, we substituted Met72 and Met100 with isosteric norleucine (Nle) to avoid possible oxidation during protein synthesis and handling. This subtle modification (S is replaced with CH_2_) is expected to have no effect on the structure and function of the protein as has been shown by numerous previous studies (see ESI†).

All peptide syntheses were performed using standard stepwise Fmoc-SPPS (ESI and Fig. S2–S5†). SELM(78–106)(A78Sez)-COSR and SELM(48–77)(U48Sez)-COSR were synthesized first as C-terminal thioester surrogates using the *N*-acylurea method,^66,67^ and then converted to thioesters following cleavage from resin. Recently, we (and others) were excited to find that the radical quencher, sodium ascorbate, completely inhibits the undesired deselenization (or desulfurization) reaction that occurs in the presence of TCEP, a commonly used reductant in NCL reactions.^44,68^ Therefore, all Sec-NCL reactions for SELM synthesis were performed in a buffer containing both TCEP and sodium ascorbate.

**Fig. 1:**
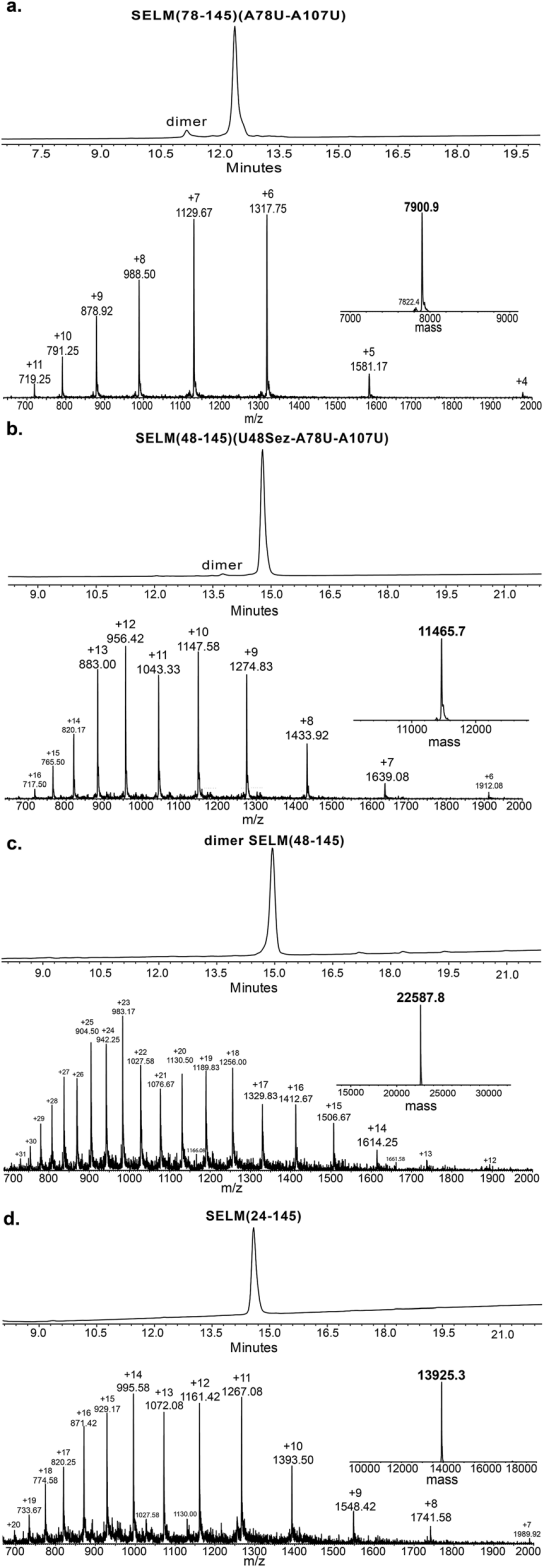
Total chemical synthesis of mature human SELM. (a–d) Analytical HPLC and ESI-MS spectra of each purified ligated product. (a) The 1^st^ purified intermediate SELM(78–145)(A78U–A107U) oxidized with Se–Se bond (obs. 7900.9 ± 0.8 Da, calc. 7899.6 Da), small amount of the protein elutes as a dimer. (b) The 2^nd^ purified intermediate SELM(48–145)(U48Sez–A78U–A107U) (obs. 11 465.7 ± 1.5 Da, calc. 11 464.7 Da). (c) The 3^rd^ purified intermediate SELM(48–145) which elutes as dimer (obs. 22 587.8 ± 1.9 Da, calc. 22 587.5 Da). (d) Mature SELM with S–Se bond in its CXXU motif (obs. 13 925.3 ± 1.5 Da, calc. 13 925.7 Da).

Under the aforementioned conditions, the ligation between SELM(78–106)(A78Sez)-COSR and SELM(107–145)(A107U) was completed in 6 h (Fig. S6†). To convert Sez to Sec, the crude mixture was then treated overnight with MeONH_2_ at pH 4–5, affording SELM(78–145)(A78U–A107U) in 43% yield (9 mg). Prior to purification, the solution was treated with a mixture of TCEP and sodium ascorbate to reduce any Se–Se bonds and simplify product isolation (Fig. 1a). The second ligation between SELM(48–77)(U48Sez)-COSR and SELM(78–145)(A78U–A107U) was completed in 4 h (Fig. S7†), providing SELM(48–145)(U48Sez–A78U–A107U) in 38% yield (5 mg) (Fig. 1b).

Under anaerobic conditions, the deselenization^34,44^ of SELM(48–145) (U48Sez–A78U–A107U) using TCEP in the presence of DTT was completed in 24 h (Fig. S8†). This is the first report for the deselenization of two Sec residues in the presence of N-terminal Sez unit in a protein. Interestingly, only insignificant amounts (<5%) of a side-product with three deselenizations (11 215 Da) was observed, indicating that the Sez was partly opened under deselenization reaction conditions (a proposed mechanism for Sez opening followed by deselenization is shown in Scheme S1†). Even though this minor side-product is unable to participate in the next Sec-NCL reaction, the desired major product with two deselenizations was isolated and then treated with MeONH_2_, giving SELM(48–145) (observed as a dimer, 22 584.9 Da) (Fig. 1c) (1 mg, 20% yield for the two steps). This result is exciting since the analogous desulfurization of Cys residues in the presence of thiazolidine unit was found to be unselective and led to opening of the thiazolidine and desulfurization.^69^ The third ligation between SELM(24–47)-COSR and SELM(48–145) was fully achieved in 4 h (Fig. S9†), at which point the mature SELM(24–145) (Fig. 1d) was isolated (0.5 mg, 40% yield) and characterized (13 925.3 Da). The exact details for the syntheses and characterizations of all peptides (Fig. S2–S5†) and the ligation reactions (Fig. S6–S9†) are shown in the ESI.†

Wild type human SELW, which is 86 residues long, was prepared from two segments with a single Cys-NCL reaction (Ile36–Cys37 ligation site, Scheme 2). SELW(37–87) was prepared by standard Fmoc-SPPS, and SELW(2–36)–Nbz was synthesized using *N*-acylurea method^66^ (ESI and Fig. S10 and S11†).

**Fig. 2:**
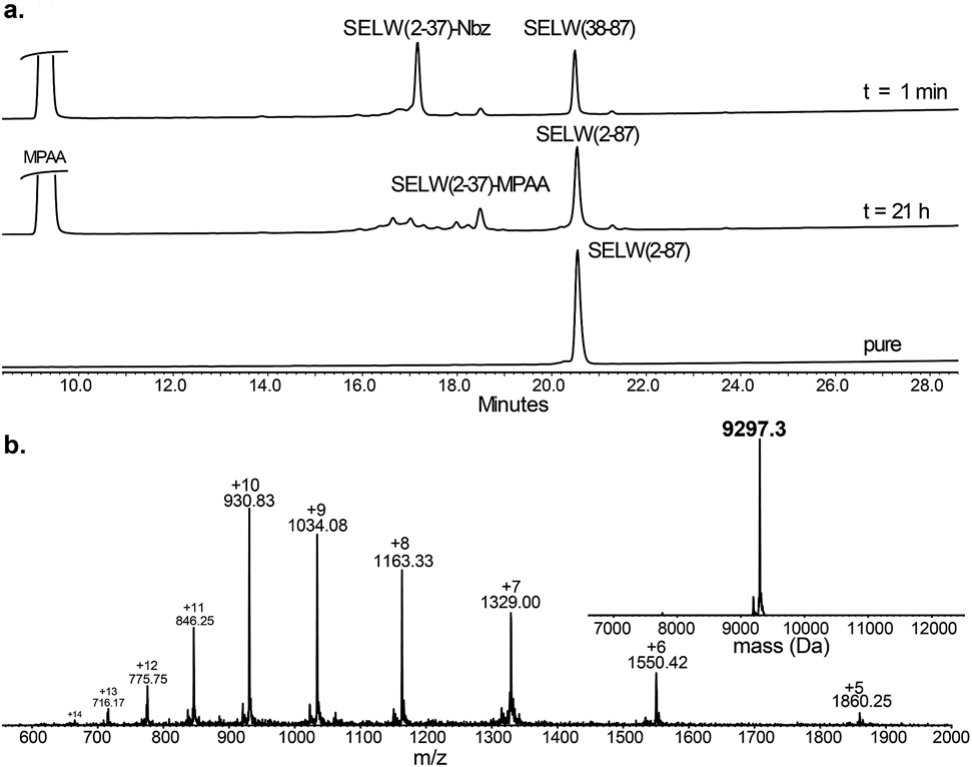
Preparation of human SELW. (a) Analytical HPLC of NCL reaction. (b) ESI-MS of SELW(2–87) oxidized with S–Se bond in its CXXU motif (obs. 9297.3 ± 0.9 Da, calc. 9296.8 Da).

Ligation between the two purified peptides (Fig. 2) was performed at 37 °C for 21 h ^70^ in the presence of MPAA^71^ and provided the wildtype human SELW in good yield (4 mg, 41% yield). Additionally, we prepared glutathionylated SELW (SELW-SG) (Fig. S12†) by treating human SELW with 6 equiv. of oxidized glutathione (GSSG) for 21 h.^33^ A glutathionylated form of SELW was isolated from rat's muscle; however, the exact role of this posttranslational modification is still unknown.^72^

**Fig. 3:**
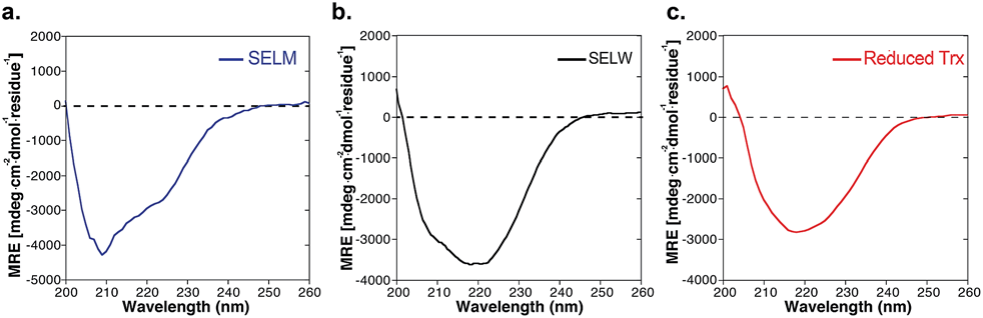
CD spectra of (a) SELM (blue); (b) SELW (black); (c) *E. coil* reduced Trx (red).

The two purified synthetic selenoproteins, human SELM and SELW, were separately dissolved in buffers to allow folding (see ESI†), upon which their structures were analyzed. CD analysis (Fig. 3a and b) shows that the two proteins are folded and contain secondary structures characteristics of the α/β fold, similar to the commercially available *E. coli* Trx (Fig. 3c), despite that Trx was in the reduced form, while SELM and SELW were oxidized.

Future efforts will aim to study the *in vitro* activity of these selenoproteins, including their redox potential, antioxidant activities,^56^ thiol-disulfide exchange activities,^50^ protein folding,^53^ and/or metal binding compatibility.^73,74^

## Conclusions

In summary, the total chemical syntheses of two natural/human selenoproteins were executed for the first time. The preparation of this family of proteins is very challenging. By using optimized Sec-NCL reactions, utilizing Sez as a protected form of Sec, and employing deselenization reactions, the chemical synthesis of this family of proteins is now in reach. This approach allows the preparation of selenoproteins in milligram quantities and in homogenous form. We believe that these results should pave the way to study these critical proteins in depth, which are currently under investigation in our research group.
