# Supplementary material for: Accessing human selenoproteins through chemical protein synthesis
Source: Chem Sci. 2016 Nov 1;8(3):1922–6. doi: 10.1039/c6sc04123j (PMC5364654; doi:10.1039/c6sc04123j)
Supplement: Supplementary file 1 [file SC-008-C6SC04123J-s001.pdf]

Electronic Supplementary Material (ESI) for Chemical Science.
This journal is © The Royal Society of Chemistry 2016

Supporting Information

Accessing Human Selenoproteins through Chemical Protein Synthesis

Linoy Dery, Post Sai Reddy, Shahar Dery#, Reem Mousa#, Orit Ktorza, Alaa Talhami and Norman Metanis*

Experimental Section:

Materials and Methods. Buffers were prepared using MilliQ water (Millipore, Merck). Ultrapure guanidinium chloride (Gn·HCl, MP Biomedicals, LLC, France) was used in all ligation reactions. $Na_2HPO_4 \cdot 12H_2O$, tris(2-carboxyethyl)phosphine hydrochloride (TCEP·HCl), ethanedithiol (EDT), triisopropylsilane (TIPS), *DL*-dithiothreitol (DTT), 2,2′-Dithiobis(5-nitropyridine) (DTNP), sodium ascorbate and 4-mercaptophenylacetic acid (MPAA) were purchased from Sigma-Aldrich (Rehovot, Israel). All Fmoc-amino acids were obtained from CS Bio Co. (Menlo Park, CA) or Matrix Innovation (Quebec City, Canada), with the following side chain protecting groups: Arg(Pbf), Asp(OtBu), Glu(OtBu), Ser(tBu), Thr(tBu), Cys(Trt), Lys(Boc), Tyr(tBu), Asn(Trt). (Pbf = 2,2,4,6,7-pentamethyl-2,3-dihydrobenzofuran-5-sulfonyl). TentaGel® R RAM resin (loading 0.18 mmol/g), Fmoc-Gly-TentaGel® R PHB resin (loading 0.2 mmol/g), and Fmoc-Leu-TentaGel® R PHB resin (loading 0.19 mmol/g) were purchased from Rapp Polymer GmbH (Germany). 1-[Bis(dimethylamino)methylene]-1H-1,2,3-triazolo[4,5-b]pyridinium 3-oxide hexafluorophosphate (HATU), 1-[Bis(dimethylamino)methylen]-5-chlorobenzotriazolium 3-oxide hexafluorophosphate, *N,N,N′,N′*-Tetramethyl-O-(6-chloro-1H-benzotriazol-1-yl)uronium hexafluorophosphate (HCTU) and Ethyl cyano(hydroxyimino)acetate (OxymaPure) were purchased from Luxembourg Biotechnologies Ltd. (Rehovot, Israel). All solvents: *N,N*-dimethylformamide (DMF), dichloromethane (DCM), acetonitrile (ACN), *N,N*-diisopropylethyl amine (DIEA), and piperidine (Pip) were purchased from Bio-Lab (Jerusalem, Israel) and were peptide synthesis, HPLC or ULC-grade. Trifluoroacetic acid (TFA) was a generous gift from Halocarbon Products (River Edge, NJ). *E. coli* Trx1 was purchased from IMCO Corp Ltd AB (Stockholm, Sweden). Fmoc-Sec(Mob)-OH and Boc-Sez-OH syntheses was reported previously.[1]

High Performance Liquid Chromatography (HPLC). Analytical reversed-phase (RP) HPLC analyses were performed on a Waters Alliance HPLC or UPLC H-Class with UV detection (220 nm and 280 nm) using a XSelect C18 column (3.5 µm, 130 Å, 4.6 × 150 mm) or XBridge C4 column (3.5 µm, 4.6 × 150 mm). Preparative and semi-preparative

RP-HPLC were performed on a Waters 150Q LC system using a XSelect C18 column (5 μm, 30 × 250 mm) or XBridge BEH300 C4 column (5 μm, 19 × 150 mm), respectively. Linear gradients of ACN (with 0.1 % TFA, buffer B) in water (with 0.1 % TFA, Buffer A) were used for all systems to elute bound peptides. The flow rates were 1 mL/min (analytical, column heated at 30 ^{o}C), 10 mL/min (semi-preparative), and 20 mL/min (C18 preparative).

Electrospray Ionization Mass Spectrometry (ESI-MS). ESI-MS was performed on LCQ Fleet Ion Trap mass spectrometer (Thermo Scientific). Peptide masses were calculated from the experimental mass to charge (*m/z*) ratios from the observed multiply charged species of a peptide. Deconvolution of the experimental MS data was performed with the help of MagTran v1.03 software.

Circular Dichroism (CD). The secondary structure content of the synthetic human SELM and SELW were compared to the commercially available *E. coli* Trx (Figure S3) using far-UV CD spectroscopy (200 to 260 nm). Spectra were recorded on J-810 spectropolarimeter (Jasco), using a quartz cuvette with a path length of 0.1 cm, and obtained by averaging 5 wavelength scans in 1 or 0.5 nm steps, with a signal averaging time of 2 s and a bandwidth of 1 nm. Each purified protein was dissolved separately in folding buffer as described bellow.

$$[\Theta]_{MER} = \frac{\Theta(mdeg)}{10 \times l(cm) \times c(M) \times N(\#amide\ bonds)}$$

SELM (final conc. 48 μM): 300 μL of 15 mM PB, 150 mM NaCl at pH 7 was used to dissolve 0.2 mg of SELM. The solution was left for one hour to allow for folding, centrifuged at 5000 rpm for 5 min, and concentration of the solution was determined by UV-Vis spectrophotometer (using theoretical $\mathcal{E}_{280\ nm}$ = 18,450 $M^{-1}\cdot cm^{-1}$).

SELW (final conc. 90 μM): 300 μL of 25 mM PB, 50 mM NaCl at pH 5.1 was used to dissolve 0.25 mg of SELW. These were the same conditions used for mouse SelW(C10S-U13C) double mutant.[2] The solution was left for one hour to allow for folding,

centrifuged at 5000 rpm for 5 min, and concentration was determined by UV-Vis spectrophotometer (using theoretical $\varepsilon_{280\ nm}$ = 5,180 $M^{-1} \cdot cm^{-1}$).

***E. coli* Trx** (final conc. 130 μM): 300 μL of 15 mM PB, 150 mM NaCl at pH 7 was used to dissolve 0.46 mg of reduced Trx. The solution was left for one hour to allow for folding and concentration was determined by UV-Vis spectrophotometer (using theoretical $\varepsilon_{280\ nm}$ = 13,980 $M^{-1} \cdot cm^{-1}$). Commercial Trx comes as a heterogeneous mixture of oxidized (S-S bond) Trx, Trx(-Met), and Trx(Met(O)). 1 mg of Trx was dissolved in 200 mM PB, 6 M Gn·HCl, pH 7 containing 0.2 M TCEP and left for 1 hour to reduce the protein. The reduced Trx was purified using analytical HPLC (XBridge C4 column, 3.5 μm, 4.6 × 150 mm) with gradient 35-70% B over 30 min.

Peptide synthesis

General procedure for Fmoc-SPPS: Peptides were prepared by automatic peptide synthesizer (CS136XT, CS Bio Inc. CA) typically on 0.25 mmol scales. Fmoc-amino acids (2 mmol in 5 mL DMF) activated with HCTU or HATU (2 mmol in 5 mL DMF) and DIEA (4 mmol in 5 mL DMF) for 5 min and allowed to couple for 25 min, with constant shaking. Fmoc-deprotection was carried out with 20% piperidine in DMF (5 min × 2). Sec and Sez were coupled using DIC/OxymaPure activation method.[1]

The sequence of mature human SELM(24-145). UniProtKB: Q8WWX9

```
24    30         40         50         60         70
ATAYRPD WNRLSGLTRA RVETCGGUQL NRLKEVKAFV TQDIPFYHNL
         80         90         100        110        120
VMKHLPGADP ELVLLGRRYE ELERIPLSEM TREEINALVQ ELGFYRKAAP
         130        140   145
DAQVPPEYVW APAKPPEETS DHADL
```

We prepared SELM(24-145) from four segments: SELM(24-47)-COSR, SELM(48-77)(U48Sez)-COSR, SELM(78-106)(A78Sez)-COSR and SELM(107-145)(A107U); by

three subsequent Sec-NCL reactions. The ligation sites are in bold and underlined. In the protein, Met72 and Met100 (in *red*) were replaced with norleucine (Nle) to avoid oxidation during synthesis and handling.[3-9] The detailed syntheses of the segments as well as the ligation reactions are described below.

Synthesis of SELM(107-145)(A107U)

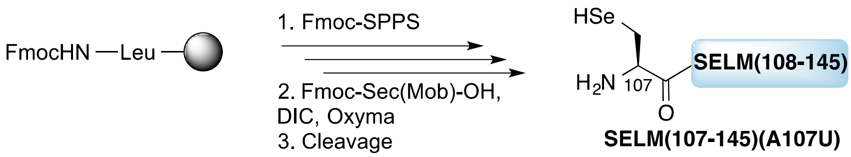

The synthesis of SELM(107-145)(A107U) was carried out on Fmoc-Leu-TentaGel®R PHB resin (0.19 mmol/g, 0.25 mmol scale) on automated peptide synthesizer. Sec107 was manually doubly coupled for 2 h (3.0 equiv Fmoc-Sec(Mob)-OH activated on ice for 5 min using 3.0 equiv OxymaPure and 2.9 equiv DIC).

Deprotection and cleavage: The resin was washed with DMF, DCM and dried under vacuum. The peptide was cleaved in the presence of 2 equiv of DTNP,[10] using TFA:water:thioanisole (95:2.5:2.5) cocktail for 5 h. The cleavage mixture was filtered and TFA was evaporated with N_2 bubbling to minimum volume, to which 8-fold volume of cold ether was added dropwise. The precipitated crude peptide was centrifuged (5000 rpm, 10 min), ether was removed and the crude peptide dissolved in ACN-water (1:1) containing 0.1% TFA and was further diluted to ~25% ACN with water and lyophilized.

Purification of SelM(107-145)(A107U): 100 mg crude peptide were dissolved in 25% ACN in water containing 0.1% TFA and purified by prep RP-HPLC (XSelect C18 column, 5 μm, 30 × 250 mm) using a gradient of 28-48% B over 50 min, to give the segment SelM(107-145)(A107U) in ~12% yield. The UPLC analysis (Figure S2) was carried out on a C18 analytical column (ACQUITY UPLC C18 column, 1.7 μm, 130 Å, 2.1 × 100 mm), using a gradient of 5% B over 3 min then 5-70% B over 7 min.

Synthesis of SELM(78-106)(A78Sez)-COSR

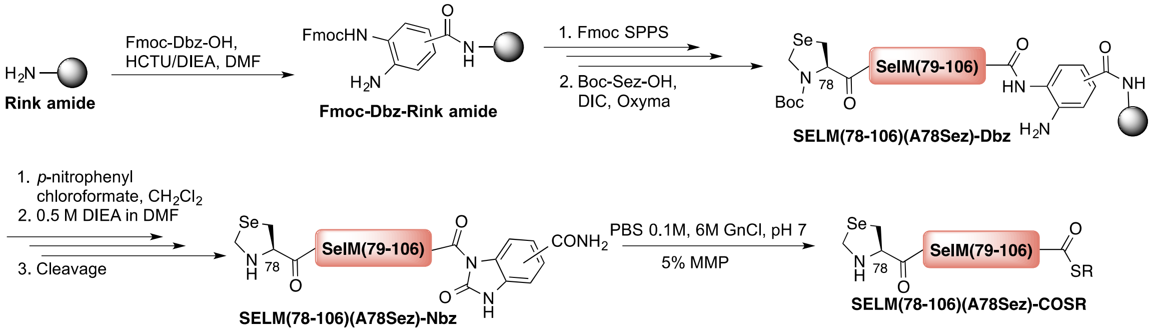

The SELM(78-106)(A78Sez)-COSR was synthesized first on Fmoc-Dbz-resin (0.25 mmol scale) on automated peptide synthesizer. Mono-Fmoc-3,4-diaminobenzoic acid (Fmoc-Dbz-OH, 3 equiv)[11] activated with HCTU (3 equiv)/DIEA (6 equiv) in DMF was doubly coupled to the free amine of TentaGel® R RAM resin (0.18 mmol/g, 0.25 mmol scale) for 1 h. Met100 was replaced with Nle, and Sez78 was manually doubly coupled for 2 h (3.0 equiv Boc-Sez-OH activated on ice for 5 min using 3.0 equiv OxymaPure and 2.9 equiv DIC).

On-resin Nbz formation: After synthesis completion, the resin was washed with DCM and a solution of *p*-nitrophenyl chloroformate (5 equiv) in DCM (10 mL/0.125 mmol resin) was added, shaken for 1 h at room temp and washed with DCM (3 × 5 mL) and DMF (3 × 5 mL). This step was repeated one more time. Next, the resin was washed with DCM and DMF, and 5 mL of 0.5 M DIEA in DMF was added and shaken for additional 30 min to complete Nbz formation (repeated twice), and washed with DMF (3 × 5 mL).

Deprotection and cleavage: The resin was washed with DMF, DCM and dried under vacuum. The peptide was cleaved using TFA:triisopropylsilane (TIPS):H_2O (95:2.5:2.5) cocktail for 5 h at room temp. The cleavage mixture was filtered and TFA was evaporated by N_2 bubbling to minimum volume, to which 8-fold volume of cold ether was added dropwise. The precipitated crude peptide was centrifuged, ether was removed and the crude peptide dissolved in ACN-water (1:1) containing 0.1% TFA and was further diluted to ~25% ACN with water and lyophilized.

Procedure for thioesterification: The crude peptide-Nbz (100 mg, ~3 mM) was dissolved in phosphate buffer (100 mM, 6 M Gn·HCl, pH ~7) and treated with methyl 3-mercaptopropionate (MMP) (5% v/v) for 5-7 h at room temp. The reaction was monitored by analytical HPLC (XSelect C18 column, 3.5 µm, 130 Å, 4.6 × 150 mm), and ESI-MS.

Purification of SelM(78-106)(A78Sez)-COSR: Upon thioesterification completion, ~0.3 mL 0.1% TFA in H_2O was added to the solution, filtered with 0.45 µm and purified by prep RP-HPLC (XSelect C18 column, 5 µm, 130 Å, 30 × 250 mm), using a gradient of 28-35% B over 50 min to give SelM(78-106)(A78Sez)-COSR in 10% yield. The UPLC analysis (Figure S3) was carried out on a C18 analytical column (ACQUITY UPLC C18 column, 1.7 µm, 130 Å, 2.1 × 100 mm), using a gradient of 5% B over 3 min then 5-70% B over 7 min).

Synthesis of SELM(48-77)(U48Sez)-COSR

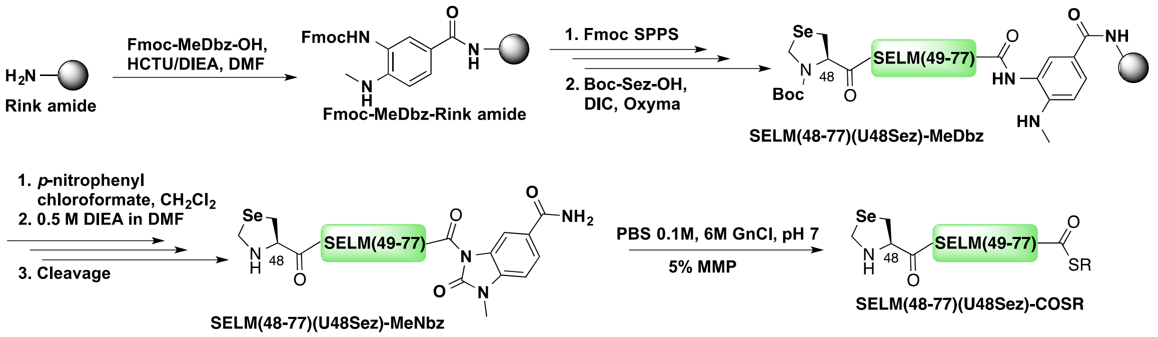

Because the C-terminal amino acid in this peptide segment is Gly, the synthesis of SELM(48-77)(U48Sez)-COSR segment was carried out on Fmoc-MeDbz-resin (0.25 mmol scale),[12] on automated peptide synthesizer. Fmoc-3-amino-4-(methylamino)benzoic acid (Fmoc-MeDbz-OH, 3 equiv) activated with HCTU (3 equiv)/DIEA (6 equiv) in DMF was manually doubly coupled to the free amine TentaGel® R RAM resin (0.18 mmol/g, 0.25 mmol scale) for 1 h. Met72 was replaced with Nle and Sez48 was manually doubly coupled for 2 h as described earlier.

On-resin MeNbz formation, deprotection and cleavage and thioesterification: MeNbz formation on this resin, deprotection and cleavage and thioesterification were performed as with SELM(78-106)(A78Sez)-Nbz peptide.

Purification of SELM(48-77)(U48Sez)-COSR: Upon thioesterification completion, ~0.3 mL 0.1% TFA in H_2O was added to the solution, filtered by 0.45 µm and purified by prep RP-HPLC (XSelect C18 column, 5 µm, 130 Å, 30 × 250 mm) using a gradient of 28-48% over 50 min to give SELM(48-77)(U48Sez)-COSR (Figure S4) in 10% yield. The UPLC analysis (Figure S4) was carried out on a C18 analytical column (3.5 µm, 130 Å, 4.6 × 150 mm), using a gradient of 5% B over 3 min then 5-70% B over 20 min.

Synthesis of SELM(24-47)-COSR

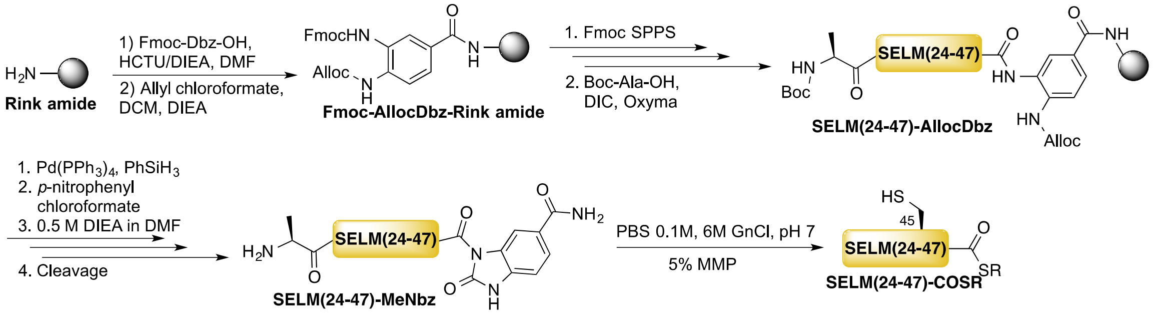

Because the two residues at the C-terminal of this peptide segment are Gly, the synthesis of SELM(24-47)-COSR was carried out on Fmoc-Dbz(Alloc)-resin (0.25 mmol scale) on automated peptide synthesizer.[13] Mono-Fmoc-3,4-diaminobenzoic acid (Fmoc-Dbz-OH, 3 equiv) activated with HCTU (3 equiv)/DIEA (6 equiv) in DMF was manually coupled doubly to the free amine TentaGel® R RAM resin (0.18 mmol/g, 0.25 mmol scale) for 1 h.

Alloc protection: The Fmoc-Dbz-resin was washed with DMF and DCM, and 0.35 M Allylchloroformate and DIEA (1 equiv to resin loading) in DCM were added to the resin and mixed for 24 h at room temperature.[14]

Standard Fmoc-SPPS was followed and finally, Boc-Ala-OH was manually doubly coupled (3.0 equiv Boc-Ala-OH, 3.0 equiv HATU and 2.9 equiv DIEA).

Alloc deprotection: The Alloc protected peptide-resin was washed and swollen in DCM for 30 min and briefly sparged with Ar. The $PhSiH_3$ (20 equiv) and $Pd(PPh_3)_4$ (0.35 equiv) in 5 mL DCM was added and mixed for 3 h.[14] The resin was washed with 0.5% DIEA in DMF, 20 mM sodium diethyldithiocarbamate trihydrate in DMF, DMF and DCM (three times each). This deprotection was repeated twice and checked by analytical HPLC and ESI-MS.

On-resin Nbz formation, deprotection and cleavage, and thioesterification: identical procedures were performed as with SELM(78-106)(A78Sez)-COSR segment.

Purification of SELM(24-47)-COSR: Upon thioesterification completion, ~0.3 mL 0.1% TFA in H_2O was added to the solution, filtered by 0.45 μm and purified by prep RP-HPLC (XSelect C18 column, 5 μm, 130 Å, 30 × 250 mm) using a gradient of 25-45% B over 42 min to give SELM(24-47)-COSR in 13% yield. The UPLC analysis (Figure S5) was carried out on a C18 analytical column (3.5 μm, 130 Å, 4.6 × 150 mm), using a gradient of 5% B over 3 min then 5-70% B over 20 min.

1st ligation reaction

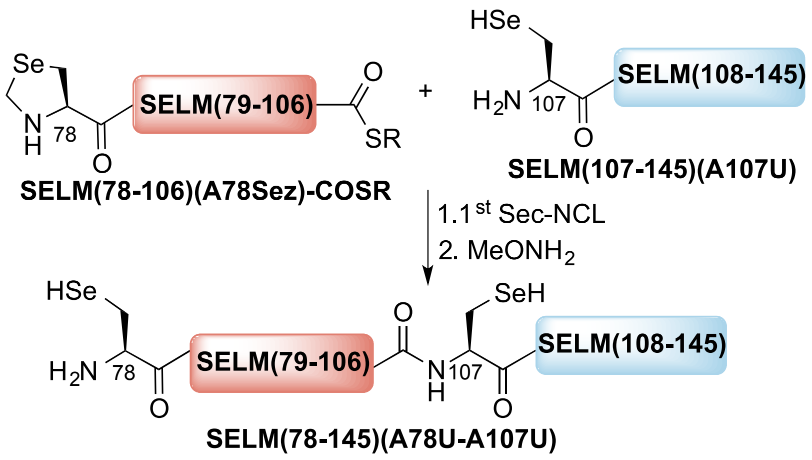

SELM(78-145)(A78U-A107U): SELM(78-106)(A78Sez)-COSR peptide (10 mg, 2.7 μmol, ~1 mM) was dissolved in 2.7 mL of argon degassed buffer (100 mM PB, 6 M Gn·HCl, 0.2 M MPAA, 0.05 M TCEP and 0.1 M sodium ascorbate, pH 7.3) and this mixture was added to SELM(107-145)(A107U) peptide (~14 mg, 3 μmol, ~1 mM). The progress of the reaction (Figure S6) was followed by analytical HPLC (XBridge C4 column, 3.5 μm, 4.6 × 150 mm) with gradient of 15-60% B over 20 min. The ligation was over in 6 h. Sez opening was performed overnight by addition 0.55 mL $MeONH_2$ (2 M in H_2O) for a final concentration of 0.2 M. Before purification, TCEP and sodium ascorbate solution was added to reduce the ligated product, then product was purified by semi-preparative HPLC (XBridge BEH300 C4 column, 5 μm, 19 × 150 mm) to afford the corresponding peptide SELM(78-145)(A78U-A107U) in 43 % (9 mg) yield.

2nd ligation reaction

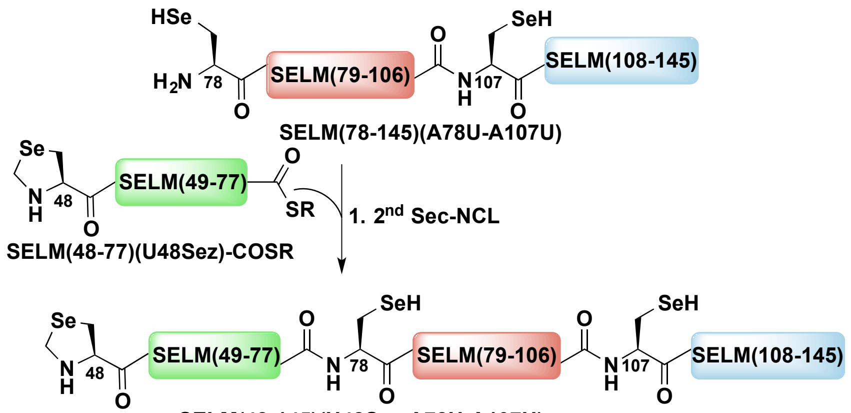

SELM(48-145)(U48Sez-A78U-A107U): SELM(48-77)(U48Sez)-COSR peptide (4 mg, 1.1 μmol, ~1 mM) was dissolved in 1.1 mL of argon degassed buffer (100 mM PB, 6 M Gn·HCl, 0.2 M MPAA, 0.05 M TCEP and 0.1 M sodium ascorbate, pH 7.3) and this mixture was added to SELM(78-145)(A78U-A107U) peptide (~9 mg, 1.1 μmol, ~1 mM). The progress of the reaction (Figure S7) was followed by analytical HPLC (XBridge C4 column, 3.5 μm, 4.6 × 150 mm) with gradient 15-60% B over 20 min. The ligation was over in 4 h. The product was purified by semi-preparative HPLC (XBridge BEH300 C4 column, 5 μm, 19 × 150 mm) to afford the corresponding peptide SELM(48-145)(U48Sez-A78U-A107U) in 38 % yield (5 mg).

Deselenization and Sez opening reactions

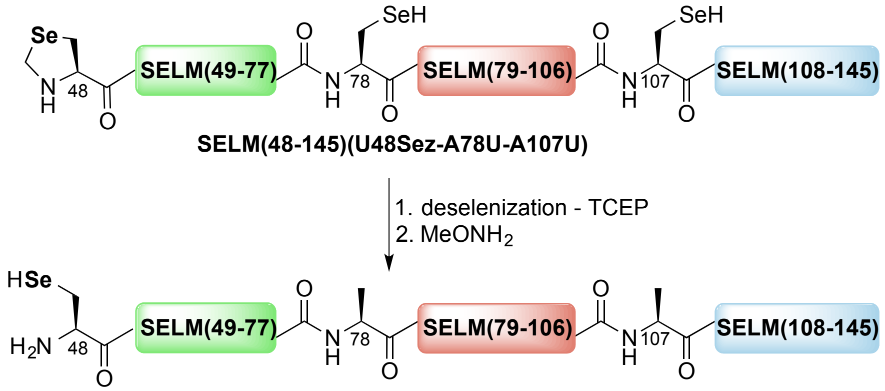

Deselenization of SELM(48-145)(U48Sez-A78U-A107U): In anaerobic chamber,

SELM(48-145)(U48Sez-A78U-A107U) (5 mg, 0.43 μmol) was dissolved in 200 μL of argon degassed buffer (100 mM PB, 6 M Gn·HCl, 100 equiv DTT, pH 7.3), and left for 30 min, upon which 100 equiv TCEP in 200 μL of the same argon degassed buffer were added.[15, 16] TCEP was added as needed (same manner) to afford completion of the deselenization. The progress of the reaction (Figure S8) was followed by analytical HPLC (XBridge C4 column, 3.5 μm, 4.6 × 150 mm) with gradient 15-60% B over 20 min. The Sez was partially opened under these conditions; hence a small peak (<5%) corresponds to side-product with three deselenizations was observed. The desired and major product was purified after 24 h by semi-preparative HPLC (XBridge C8 column, 5 μm, 10 × 150 mm) to afford the corresponding peptide SELM(48-145)(U48Sez).

SELM(48-145): SELM(48-145)(U48Sez) (2.5 mg, 0.11 μmol) was dissolved in 0.4 mL of argon degassed buffer (100 mM PB, 6 M Gn·HCl, pH 7.3) and 45 μL $MeONH_2$ (2 M in H_2O) were added and the reaction was left overnight. The product was purified as dimer with Se-Se bond by semi-preparative HPLC (C8 column) to afford 1 mg the corresponding peptide SELM(48-145) (total yield for the two steps including two purifications was 20%).

__3rd ligation reaction__

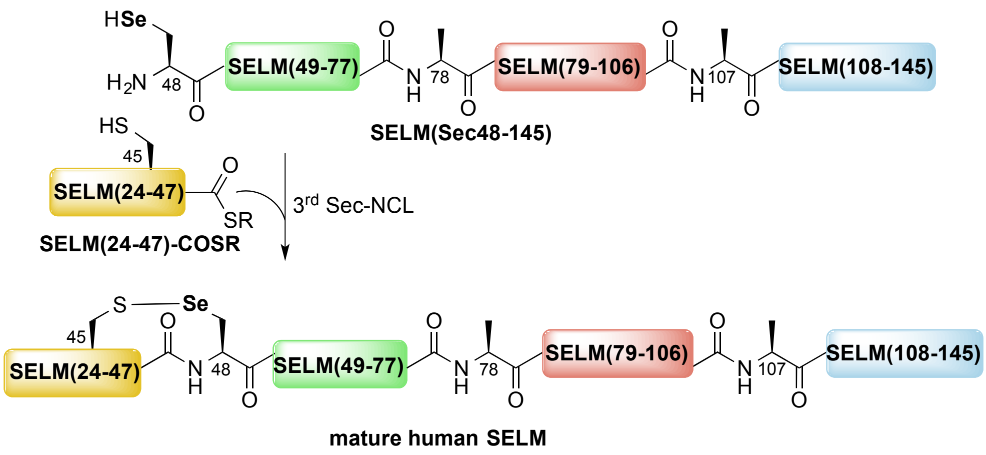

SELM(24-145). SELM(48-145) dimer peptide (1 mg, 0.044 μmol) was dissolved in 400 μL of argon degassed buffer (100 mM PB, 6 M Gn·HCl, 0.2 M MPAA, 0.05 M TCEP

and 0.1 M sodium ascorbate, pH 7.3) and this mixture was added to SELM(24-47)-COSR peptide (2 mg, 0.73 mmol), which was in excess since SELM(48-145) segment and product SELM(24-145) co-elutes at same retention time. The progress of the reaction (Figure S9) was followed by analytical HPLC (C4 column, 15-60% B over 20 min). The ligation was completed in 4 h. The product was purified by analytical HPLC (C4 column, 25-60% B over 20 min) to afford the corresponding peptide SELM(24-145) in ~40% yield (0.5 mg).

Human SELW sequence. UniProtKB: P63302

10 20 30 40 50
MALAVRVVY**C** GA**U**GYKSKYL QLKKKLEDEF PGRLD**IC**GEG TPQATGFFEV
60 70 80
MVAGKLIHSK KKGDGYVDTE SKFLKLVAAI KAALAQG

We prepared wildtype human SELW(2-87) from two peptide segments namely SELW(2-36)-Nbz and SELW(37-87) with a single Cys-NCL reaction. The ligation site is in bold and underlined. In the protein, Met51 (in *red*) was replaced by Nle to avoid oxidation. The detailed synthesis of the two peptide segments as well as the ligation reaction are described below.

Synthesis of SELW(37-87)

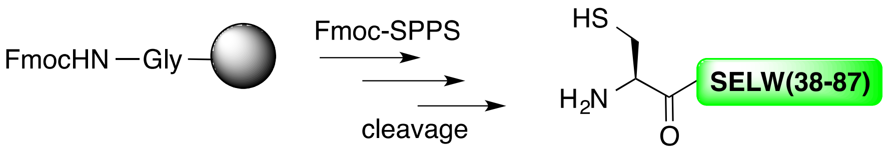

SELW(37-87)

The synthesis of SELW(37-87) was carried out on Fmoc-Gly-TentaGel® R PHB resin (0.20 mmol/g, 0.25 mmol scale) on automated peptide synthesizer. Met51 was replaced

with Nle, Gly65 and Gly54 were manually coupled as (Dmb)Gly (2.5 equiv Fmoc-(Dmb)Gly-OH,[17] 2.5 equiv HATU and 5 equiv DIEA) and Asp64 and Ala53 were doubly coupled.

Deprotection and cleavage: The resin was washed with DMF, DCM, MeOH and dried under vacuum. The peptide was cleaved using TFA:ethanedithiol (EDT):TIPS:water (94:2.5:1:2.5) cocktail for 3 h. The cleavage mixture was filtered and TFA was evaporated by N_2 bubbling to minimum volume, to which 8-fold volume of cold ether was added dropwise. The precipitated crude peptide was centrifuged, ether was removed and the crude peptide dissolved in ACN-water (1:1) containing 0.1% TFA and was further diluted to ~30% ACN with water and lyophilized.

Purification of SELW(37-87): 100 mg crude peptide were dissolved in 40% ACN in water containing 0.1% TFA and purified by prep RP-HPLC (XSelect C18 column, 5 μm, 30 × 250 mm) to give fragment SELW(37-87) in ~10% yield. The HPLC analysis (Figure S10) was carried out on analytical C18 column, with gradient 5-60% B over 30 min.

Synthesis of SELW(2-36)-Nbz

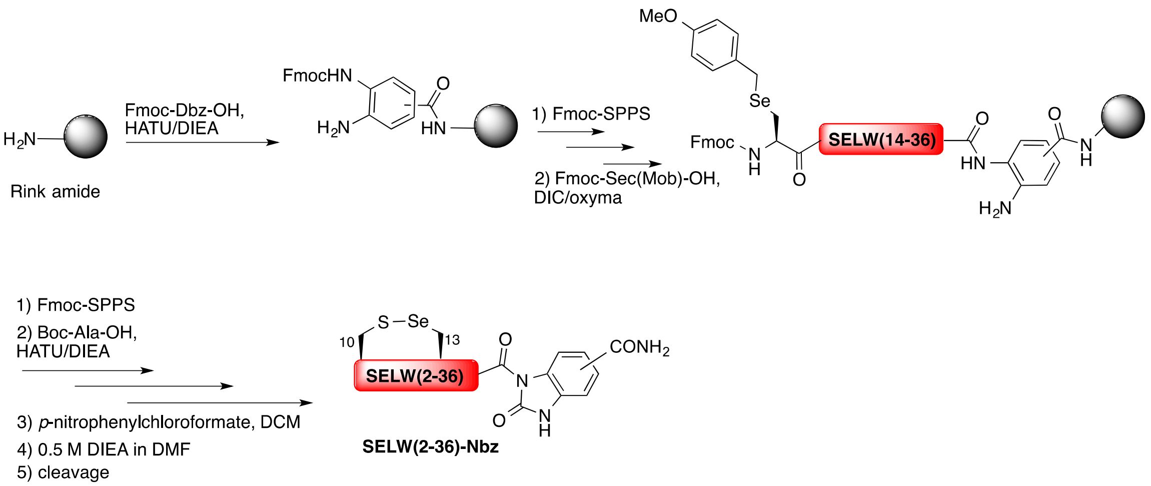

The synthesis of SELW(2-36)-Nbz was carried out first on Fmoc-Dbz-resin (0.25 mmol scale) on automated peptide synthesizer. Mono-Fmoc-3,4-diaminobenzoic acid (Fmoc-Dbz-OH, 3 equiv)[11] activated with HATU (3 equiv)/DIEA (6 equiv) in DMF was

manually doubly coupled to the free amine TentaGel® R RAM resin (0.18 mmol/g, 0.25 mmol scale) for 1 h. The first amino acid Ile36 was manually doubly coupled (5.5 equiv Fmoc-Ile-OH, 5.0 equiv HATU and 10 equiv DIEA) and Sec13 was manually doubly coupled as Sec(Mob) (3.0 equiv Fmoc-Sec(Mob)-OH, 3.0 equiv OxymaPure and 2.9 equiv DIC). Gly14 was manually coupled as (Dmb)Gly (2.5 equiv Fmoc-(Dmb)Gly-OH,[17] 2.5 equiv HATU and 5 equiv DIEA) and Arg33, Val8, Val7, Arg6, Val5, Ala4 and Leu3 were doubly coupled as well. Finally, Ala was manually doubly coupled at position 2 using Boc-Ala-OH (5.5 equiv, 5 equiv HATU and 10 equiv DIEA in DMF), reacted on 0.125 mmol resin based on the initial loading.

On-resin Nbz formation: After synthesis completion, the resin was washed with DCM and a solution of *p*-nitrophenyl chloroformate (5 equiv) in DCM (5 mL/0.125 mmol) was added, shaken for 1 h at 25 °C and washed with DCM (3 × 5 mL) and DMF (3 × 5 mL). This step was repeated one more time. Following this, the resin was washed and a solution of 0.5 M DIEA in DMF (3 mL/0.125 mmol) was added and shaken for additional 30 min to complete the cyclization and Nbz formation (repeated twice), and washed with DMF (3 × 5 mL).

Deprotection and cleavage: The resin was washed with DMF, DCM and dried under vacuum. The peptide was cleaved in the presence of 2 equiv of DTNP,[10] using TFA:EDT:TIPS:water:thioanisole (92:2.5:1:2.5:2) cocktail for 5 h. The cleavage mixture was filtered and TFA was evaporated by N_2 bubbling to minimum volume, to which 8-fold volume of cold ether was added dropwise. The precipitated crude peptide was centrifuged, ether was removed and the crude peptide dissolved in ACN-water (1:1) containing 0.1% TFA and was further diluted to ~30% ACN with water and lyophilized.

Purification of SELW(2-36)-Nbz: Crude peptide (150 mg, contains DTNP impurity) was dissolved in 25% ACN in water containing 0.1% TFA and purified by prep RP-HPLC (XSelect C18 column, 5 µm, 30 × 250 mm) to give fragment SELW(2-36)-Nbz (Figure S11) in ~20% yield. The HPLC analysis was carried out on a C18 analytical column using a gradient of 5% B over 1 min then 5-60% B over 30 min.

<u>Ligation reaction for SELW</u>

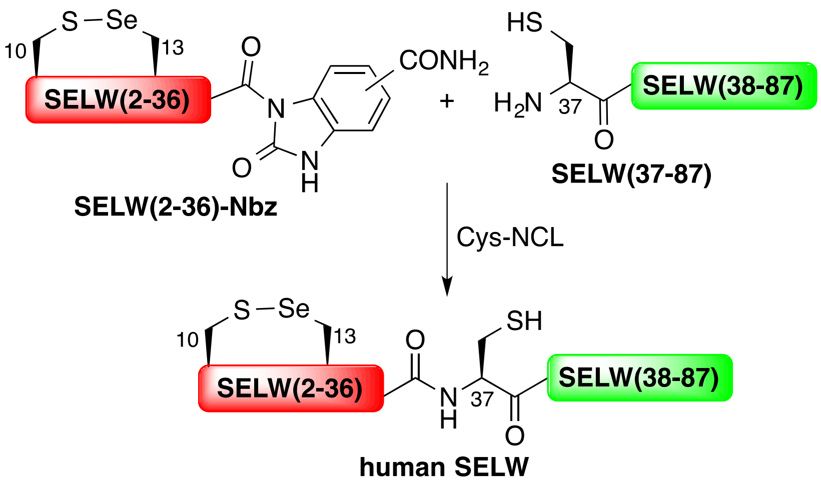

Wildtype human SELW(2-87): SELW(2-36)-Nbz peptide (5.2 mg, 1.21 μmol, ~1 mM) was dissolved in 1.06 mL of argon degassed buffer (100 mM PB, 6 M Gn·HCl, 0.2 M MPAA, pH 7.3) and this mixture was added to SELW(37-87) peptide (~5.6 mg, 1.06 μmol, 1 mM) and after 1 min at room temperature, incubated at 37 ºC for 6 h. The progress of the reaction (Figure 2) was followed by analytical HPLC (C4 column, 5% B over 1 min then 5-60% B over 30 min) (small aqueous mixture of TCEP:sodium ascorbate – 1 : 3) was added to analytical sample prior to run the analytical HPLC to reduce any Se-Se, Se-S or S-S bonds and to obtain a single peak). Additional portion of SELW(2-34)-Nbz peptide (~5.6 mg) was added and incubated at 37 ºC for overnight to complete the reaction (because SELW(37-87) segment and product SELW(2-87) co-elutes at same time points). Before purification, TCEP and sodium ascorbate solution was added to reduce the ligated product, then the product was purified by semi-preparative HPLC (C4 column) to afford the corresponding peptide SELW(2-87) in 41 % yield (4 mg).

Glutathionylated SELW, SELW-SG: SELW(2-87) protein (0.5 mg, 0.05 μmol) was dissolved in 100 μL of argon degassed Tris buffer (100 mM, 1 mM EDTA, pH 8) and this mixture was added 2 equiv of GSSG in H_2O (2.5 μL). After 5 h additional 4 equiv of GSSG in H_2O (5 μL) was added and incubated for 21 h and the progress of the reaction was followed by analytical HPLC (C4 column, 15% B over 1 min then 15-50% B over

65 min). The product was purified by semi-preparative HPLC (C8 column) to afford the corresponding peptide SELW(2-87)-SG in 41 % yield (0.2 mg) (Figure S12).

Supplementary Table, Figures and Schemes

Table S1. SELM and SELW in bold. Adapted from ref. [18]

Selenoprotein name	Sec location	Protein length
15 kDa (SEP15)	93	162
DI1	126	249
DI2	133	265
DI3	144	278
GPx1	47	201
GPx2	40	190
GPx3	73	226
GPx4	73	197
GPx6	73	221
SELH	44	122
SELI	387	397
SELK	92	94
**SELM**	**48**	**145**
SELN	428	556
SELO	667	669
SELP	59, 300, 318, 330, 345, 352, 367, 369, 376, 378	381
MsrB1	95	116
SELS	188	189
SPS2	60	448
SELT	36	182
TrxR1	498	499
TrxR2	655	656
TrxR3	522	523
SELV	273	346
**SELW**	**13**	**87**

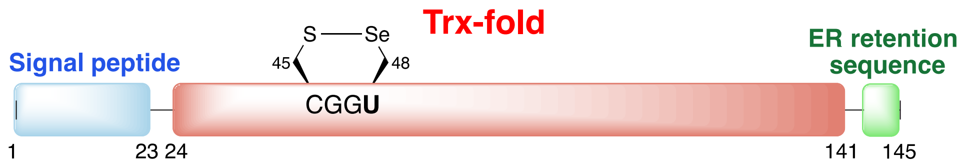

Figure S1. Schematic illustration of human SELM sequence. The N-terminal signal peptide is in *blue* (1-23), the Trx-fold in *red* (24-141) and ER-retention sequence in *green* (142-145). The active site redox motif CGGU is shown with the expected S-Se bond.

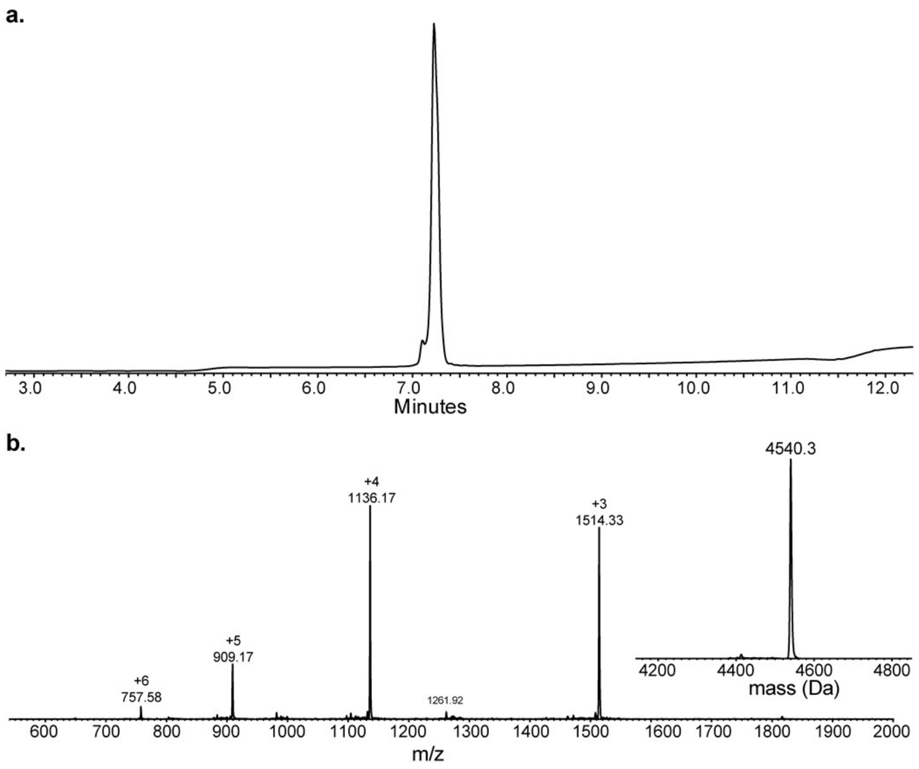

Figure S2. Characterization of purified SELM(107-145)(A107U) segment. **a.** UPLC analysis; **b**. ESI-MS with its deconvoluted mass (inset) (obs. 4540.3 ± 0.7 Da, calc. 4540.9 Da), for the TNP-adduct.

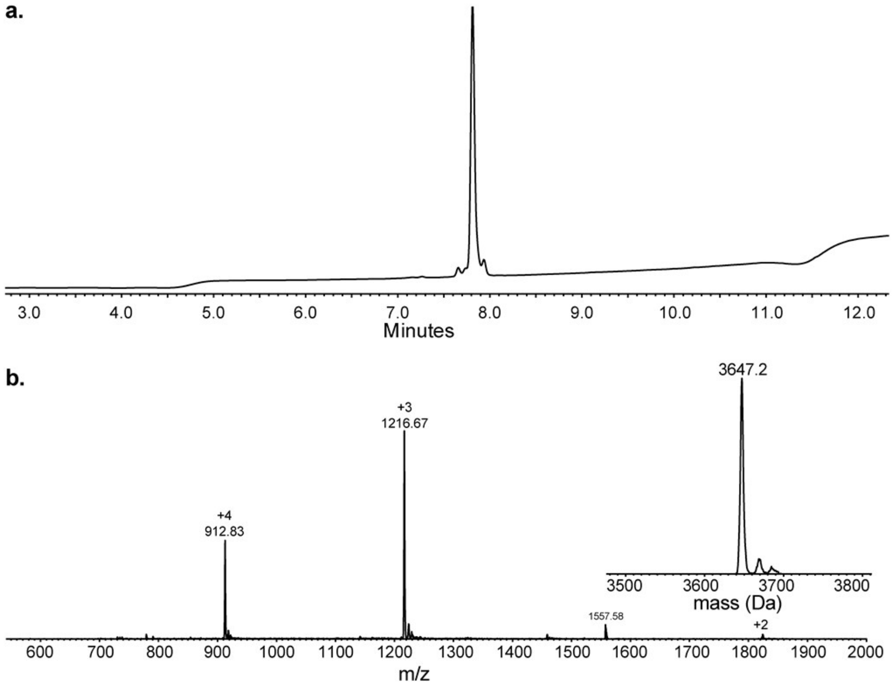

Figure S3. Characterization of purified SELM(78-106)(A78Sez)-COSR segment. **a**. UPLC analysis; **b**. ESI-MS with its deconvoluted mass (inset) (obs. 3647.2 ± 0.2 Da, calc. 3647.0 Da).

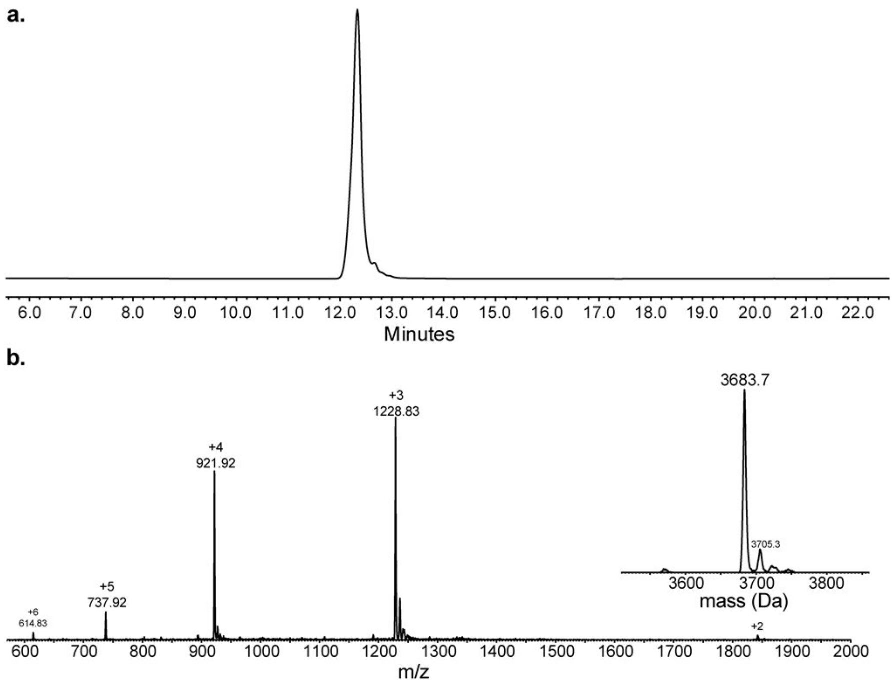

Figure S4. Analytical HPLC and ESI-MS of purified SELM(48-77)(U48Sez)-COSR

segment. **a**. HPLC analysis; **b**. ESI-MS with its deconvoluted mass (inset) (obs. 3683.7 ± 0.7 Da, calc. 3683.2 Da).

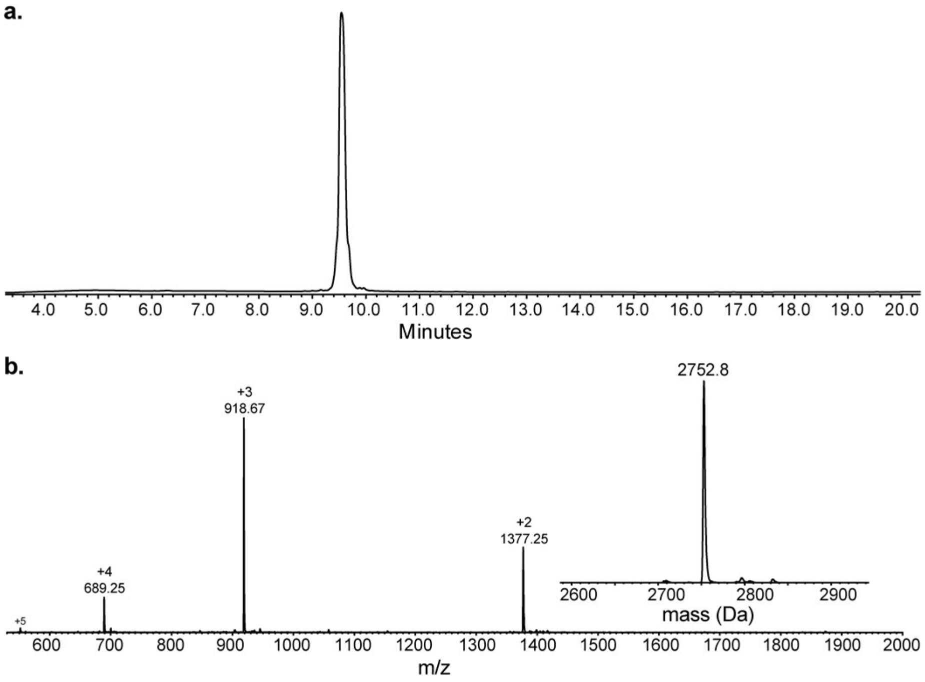

Figure S5. Characterization of purified SELM(24-47)-COSR segment. **a**. HPLC analysis; **b**. ESI-MS with its deconvoluted mass (inset) (obs. 2752.8 ± 0.3 Da, calc. 2753.1 Da).

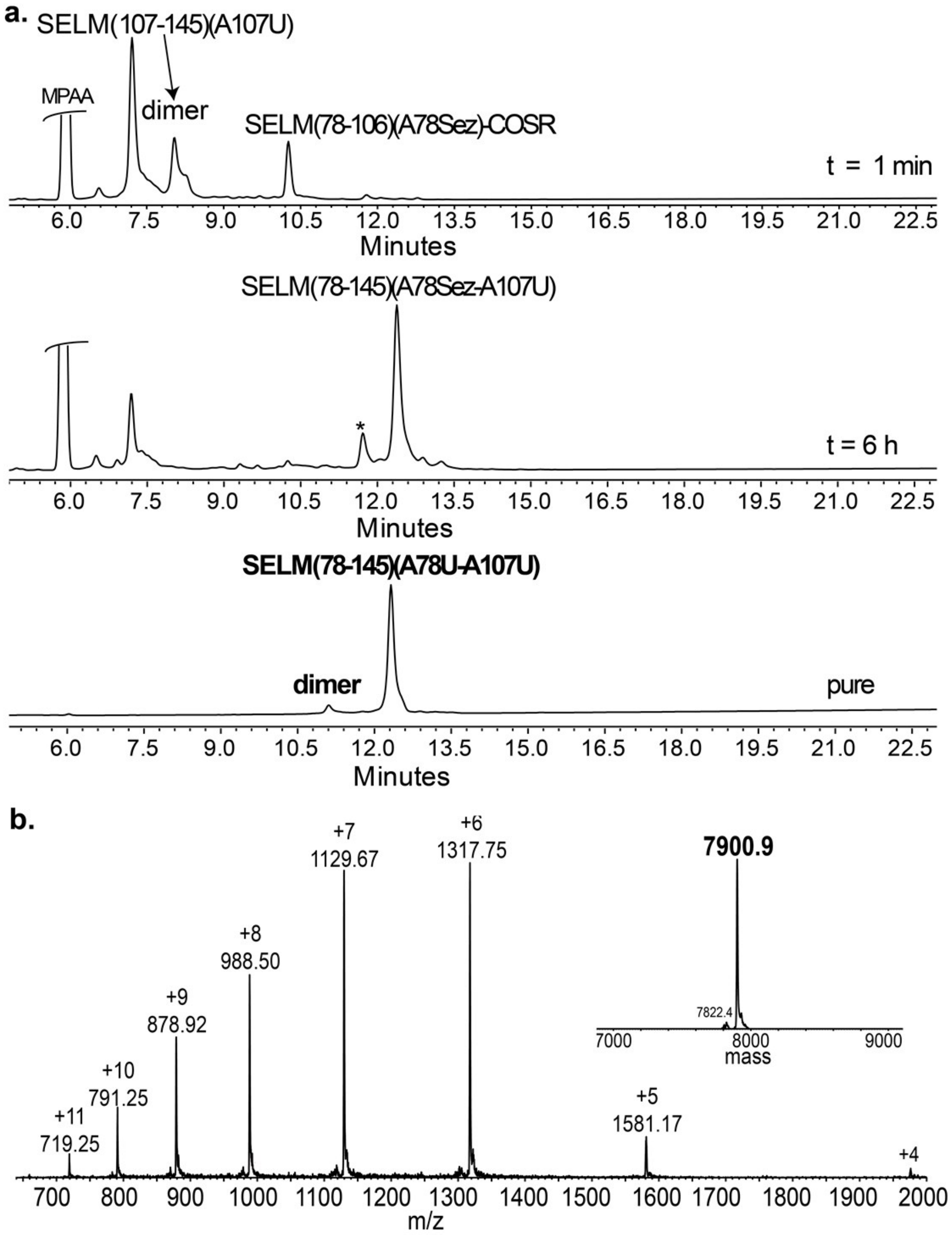

Figure S6. Preparation of SELM(78-145)(A78U-A107U). **a**. Analytical HPLC of 1st Sec-NCL reaction. The ligated product was treated overnight with $MeONH_2$ and purified by prep HPLC. **b**. ESI-MS of SELM(78-145)(A78U-A107U) oxidized with Se−Se bond (obs. 7900.9 ± 0.8 Da, calc. 7899.6 Da). Asterisks is ligated product with Se-Se bond with SELM(107-145)(A107U).

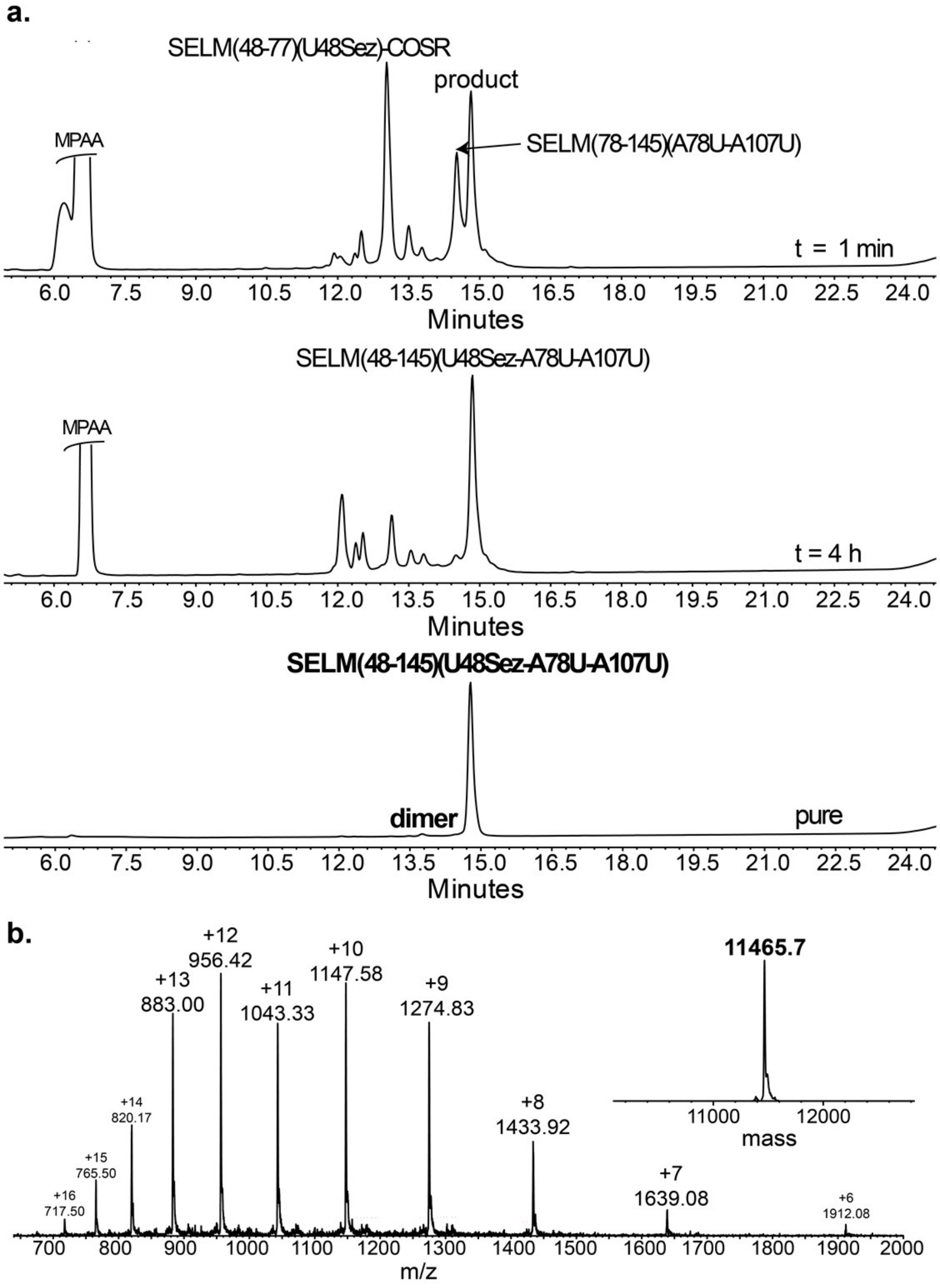

Figure S7. Preparation of SELM(48-145)(U48Sez-A78U-A107U). **a**. Analytical HPLC of the 2nd Sec-NCL reaction. The ligated product was purified by prep HPLC. **b**. ESI-MS of SELM(48-145)(U48Sez-A78U-A107U) (obs. 11465.7 ± 1.5 Da, calc. 11464.7 Da).

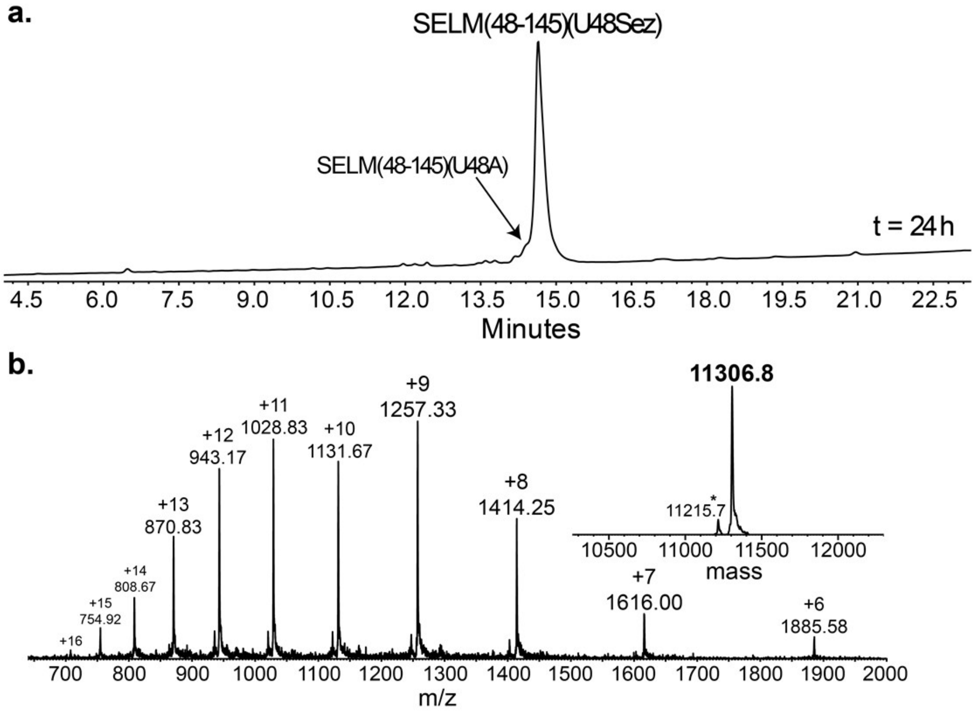

Figure S8. Deselenization of SELM(48-145)(U48Sez-A78U-A107U). **a**. Analytical HPLC of the deselenization reaction after 24 h. A negligible undesired product with three deselenizations; SELM(48-145)(U48A), is observed. **b**. ESI-MS of desired product SELM(48-145)(U48Sez) (obs. 11306.8 ± 1.1 Da, calc. 11306.8 Da). Asterisks shows the mass of the SELM(48-145)(U48A).

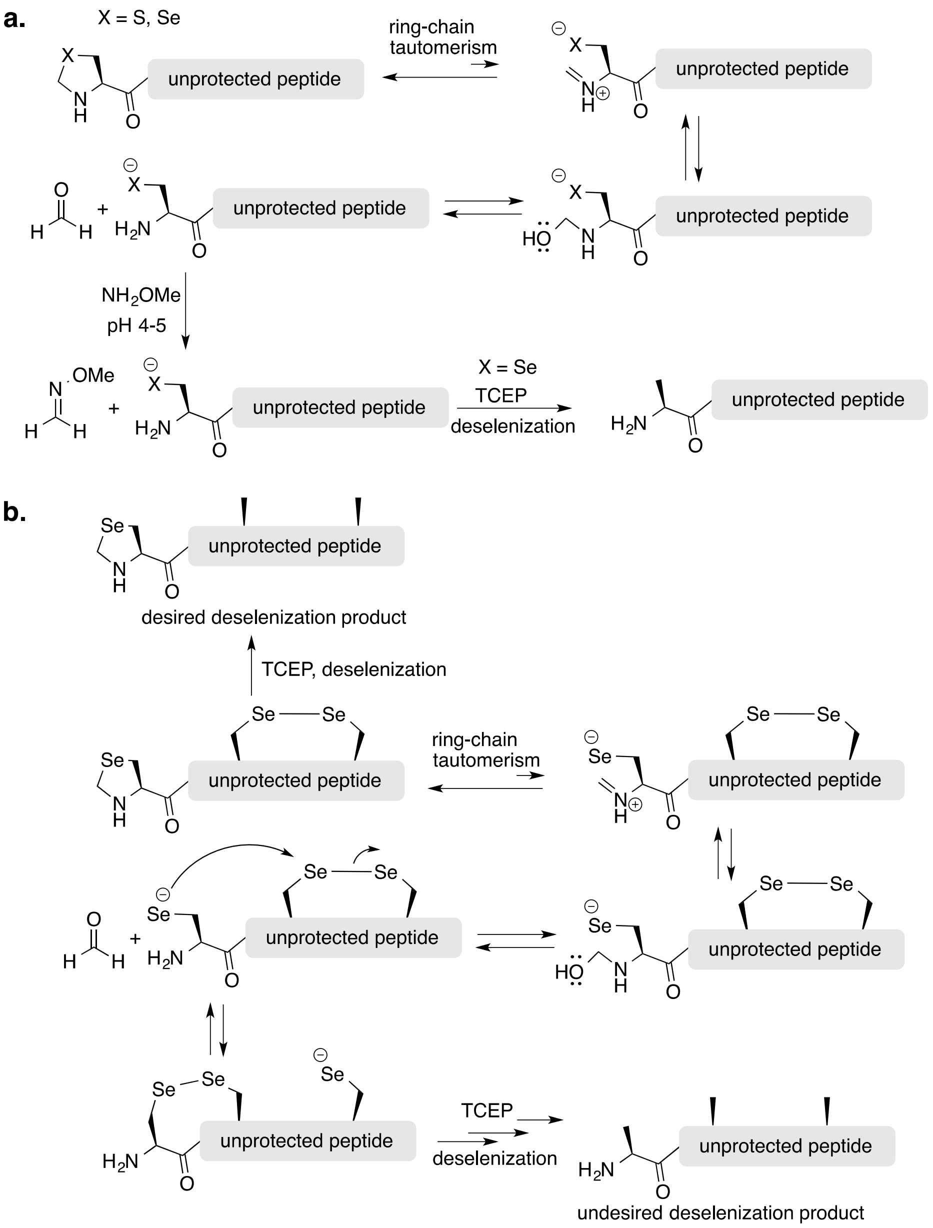

Scheme S1. Proposed mechanisms for the opening of N-terminal Thz and Sez containing peptides in aqueous solution, based on previous and recent work.[19-22] **a**. Opening in the presence of $MeONH_2$, which traps the released formaldehyde forming oxime, upon which deselenization by TCEP provides the desired N-terminal Ala. **b**. If the peptide with N-terminal Sez contains also other Sec residues, the released N-terminal Sec can be “trapped” as diselenide, either by selenol-diselenide exchange reactions or simple oxidation by molecular O_2. Therefore the deselenization of this peptide can be unselective providing undesired product with all Sec residues converted to Ala.

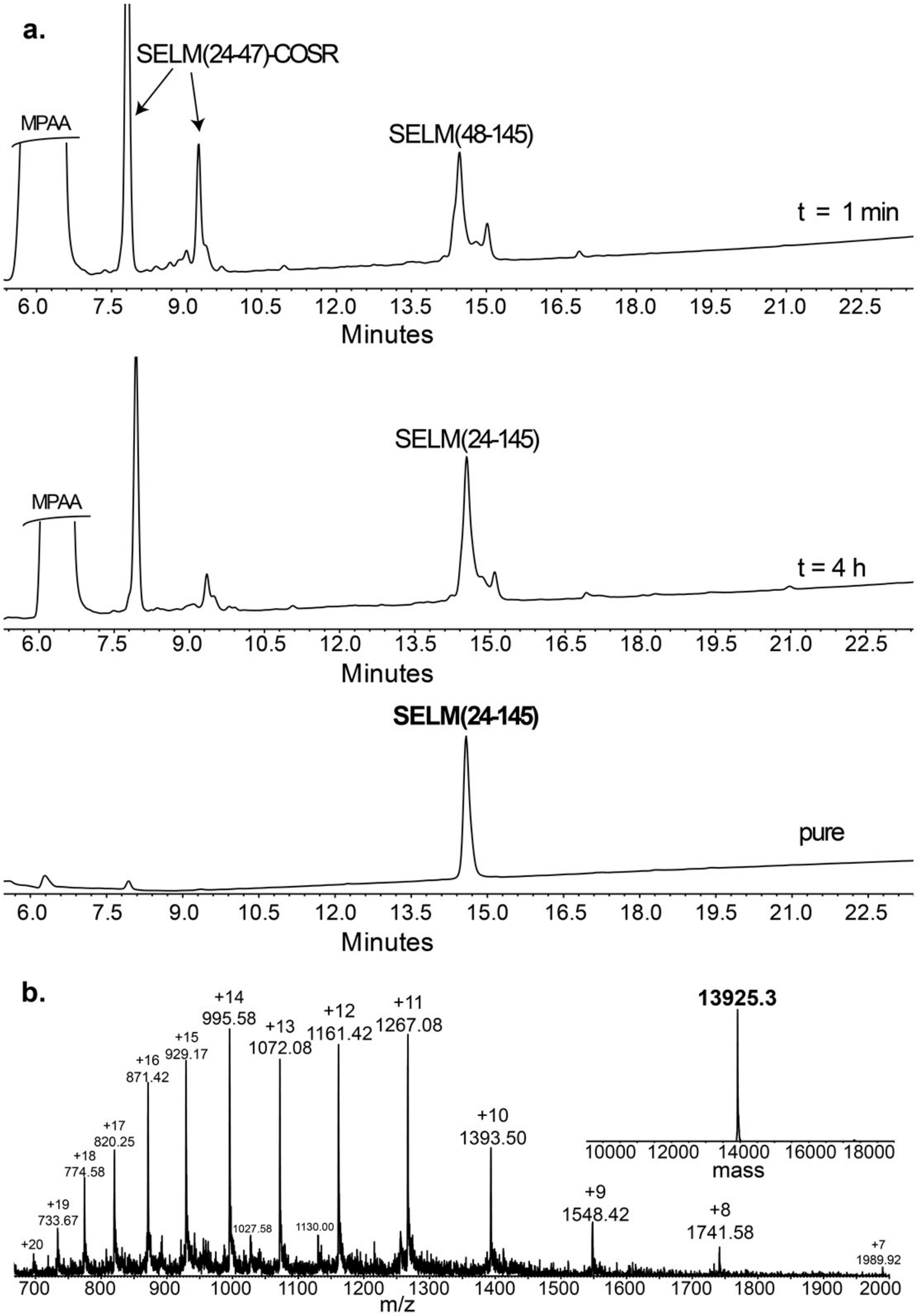

Figure S9. Preparation of mature human SELM(24-145). **a**. Analytical HPLC of the 3rd Sec-NCL reaction. **b**. ESI-MS of SELM(24-145) oxidized with S−Se bond in the active site motif ^{45}CXX^{48}U (obs. 13925.3 ± 1.6 Da, calc. 13925.7 Da).

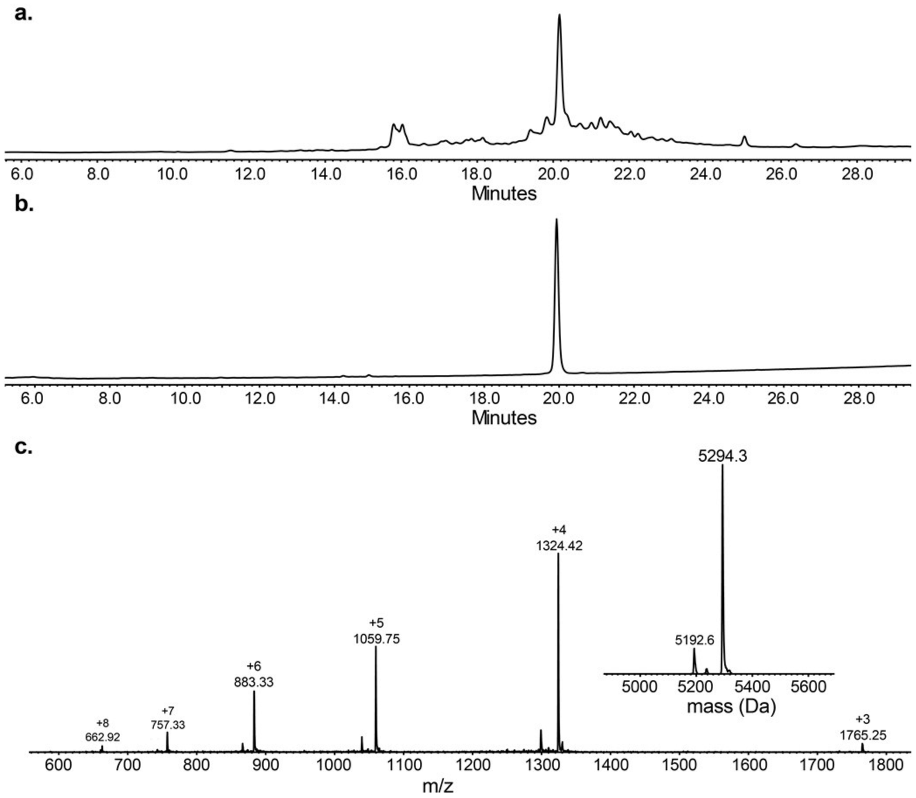

Figure S10. Analytical HPLC and ESI-MS of SELW(37-87) segment. HPLC analysis of **a**. crude and **b**. purified SELW(37-87); **c**. ESI-MS of purified SELW(37-87), with its deconvoluted mass (inset) (obs. 5294.0±0.7 Da , calc. 5294.2 Da).

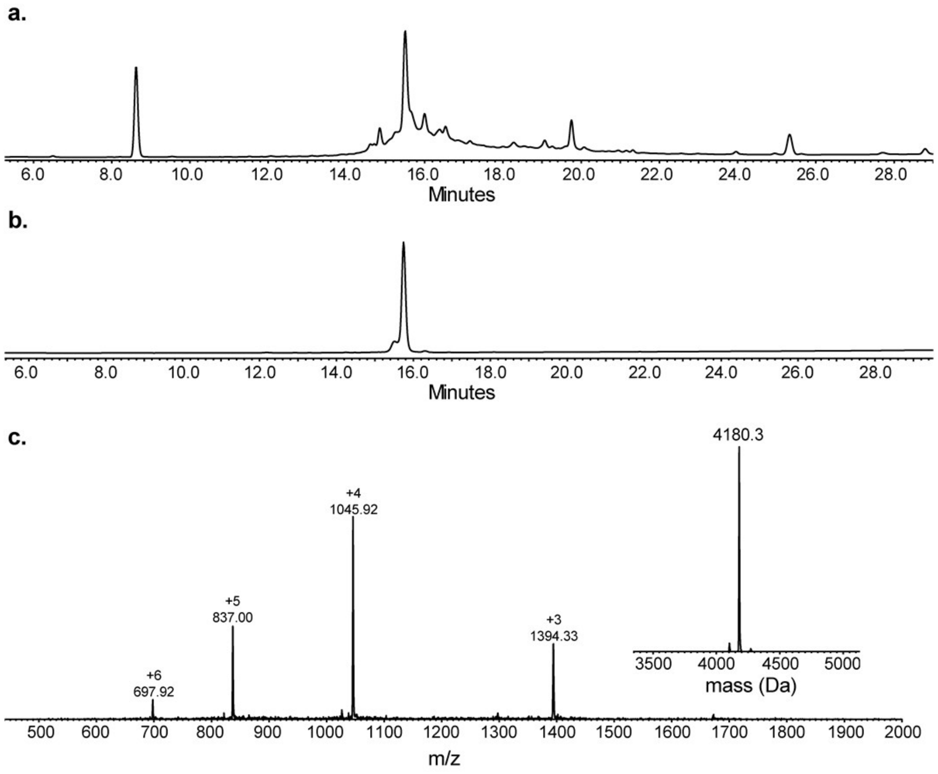

Figure S11. Analytical HPLC and ESI-MS of SELW(2-36)-Nbz segment. HPLC analysis of **a**. crude and **b**. purified SELW(2-36)-Nbz; **c**. ESI-MS of purified SELW(2-36)-Nbz, with its deconvoluted mass (inset) (obs. 4180.3 ± 0.8 Da , calc. 4179.8 Da) oxidized with Se−S bond.

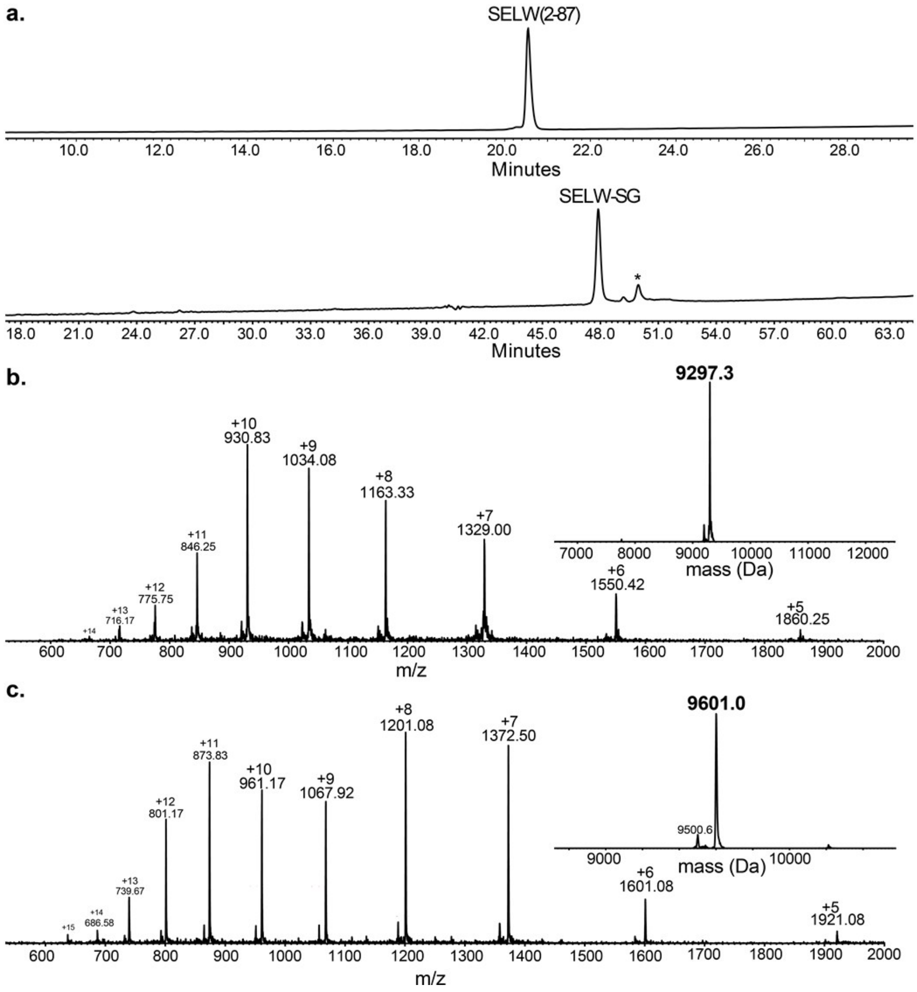

Figure S12. Preparation of glutathionylated SELW, SELW-SG. **a**. Analytical HPLC of pure SELW and SELW-SG, asterisks is unreactive SELW starting material (note that different gradient was used in order to isolate the product SELW-SG from unreactive SELW). **b**. ESI-MS of SELW(2-87) oxidized with Se−S bond (obs. 9297.3 ± 0.9 Da, calc. 9296.8 Da). **c**. ESI-MS of SELW-SG (obs. 9601.0 ± 1.3 Da, calc. 9602.1 Da).

References

1. P. S. Reddy, S. Dery and N. Metanis, *Angew Chem. Int. Ed.*, 2016, **55**, 992-995.
2. F. L. Aachmann, D. E. Fomenko, A. Soragni, V. N. Gladyshev and A. Dikiy, *J. Biol. Chem.*, 2007, **282**, 37036-37044.
3. N. Metanis, A. Brik, P. E. Dawson and E. Keinan, *J. Am. Chem. Soc.*, 2004, **126**, 12726-12727.
4. N. Metanis and D. Hilvert, *Angew. Chem. Int. Ed.*, 2012, **51**, 5585-5588.
5. C. Wolschner, A. Giese, H. A. Kretzschmar, R. Huber, L. Moroder and N. Budisa, *Proc. Natl. Acad. Sci. USA*, 2009, **106**, 7756-7761.
6. S. Roosenburg, P. Laverman, L. Joosten, A. Eek, W. J. G. Oyen, M. de Jong, F. P. J. T. Rutjes, F. L. van Delft and O. C. Boerman, *Bioconjugate Chem*, 2010, **21**, 663-670.
7. S. K. Maity, G. Mann, M. Jbara, S. Laps, G. Kamnesky and A. Brik, *Org. Lett.*, 2016, **18**, 3026-3029.
8. S. K. Maity, M. Jbara, S. Laps and A. Brik, *Angew. Chem. Int. Ed.*, 2016, **55**, 8108-8112.
9. F. K. Deng, L. Zhang, Y. T. Wang, O. Schneewind and S. B. H. Kent, *Angew. Chem. Int. Ed.*, 2014, **53**, 4662-4666.
10. S. Flemer and R. J. Hondal, *Biopolymers*, 2011, **96**, 439-439.
11. J. B. Blanco-Canosa and P. E. Dawson, *Angew. Chem. Int. Ed.*, 2008, **47**, 6851-6855.
12. J. B. Blanco-Canosa, B. Nardone, F. Albericio and P. E. Dawson, *J. Am. Chem. Soc.*, 2015, **137**, 7197-7209.
13. S. K. Mahto, C. J. Howard, J. C. Shimko and J. J. Ottesen, *Chembiochem*, 2011, **12**, 2488-2494.
14. N. Thieriet, J. Alsina, E. Giralt, F. Guibe and F. Albericio, *Tet. Lett.*, 1997, **38**, 7275-7278.
15. S. Dery, P. S. Reddy, L. Dery, R. Mousa, R. Notis Dardashti and N. Metanis, *Chem. Sci.*, 2015, **6**, 6207-6212.
16. N. Metanis, E. Keinan and P. E. Dawson, *Angew. Chem. Int. Ed.*, 2010, **49**, 7049-7053.
17. P. t. Hart, L. H. J. Kleijn, G. de Bruin, S. F. Oppedijk, J. Kemmink and N. I. Martin, *Org. Biomol. Chem.*, 2014, **12**, 913-918.
18. G. V. Kryukov, S. Castellano, S. V. Novoselov, A. V. Lobanov, O. Zehtab, R. Guigo and V. N. Gladyshev, *Science*, 2003, **300**, 1439-1443.
19. R. G. Kallen, *J. Am. Chem. Soc.*, 1971, **93**, 6227-6235.
20. J. J. Pesek and J. H. Frost, *Tetrahedron*, 1975, **31**, 907-913.
21. F. Fülöp, J. Mattinen and K. Pihlaja, *Tetrahedron*, 1990, **46**, 6545-6552.
22. M. Jbara, S. K. Maity, M. Seenaiah and A. Brik, *J. Am. Chem. Soc.*, 2016, **138**, 5069-5075.
